# Targeting Alzheimer’s Disease: Evaluating the Efficacy of C-1 Functionalized N-Aryl-Tetrahydroisoquinolines as Cholinergic Enzyme Inhibitors and Promising Therapeutic Candidates

**DOI:** 10.3390/ijms25021033

**Published:** 2024-01-14

**Authors:** Dunja Jovanović, Ana Filipović, Goran Janjić, Tamara Lazarević-Pašti, Zdravko Džambaski, Bojan P. Bondžić, Aleksandra M. Bondžić

**Affiliations:** 1 Vinča Institute of Nuclear Sciences, National Institute of the Republic of Serbia, University of Belgrade, P.O. Box 522, 11000 Belgrade, Serbia; dunja.jovanovic@vin.bg.ac.rs (D.J.); tamara@vin.bg.ac.rs (T.L.-P.); 2 Institute of Chemistry, Technology and Metallurgy, National Institute of the Republic of Serbia, University of Belgrade, Njegoševa 12, 11000 Belgrade, Serbia; ana.filipovic@nanosys.ihtm.bg.ac.rs (A.F.); goran.janjic@ihtm.bg.ac.rs (G.J.); zdravko.dzambaski@ihtm.bg.ac.rs (Z.D.)

**Keywords:** tetrahydroisoquinolines, acetylcholinesterase, butyrylcholinesterase, Alzheimer’s disease

## Abstract

We have synthesized 22 C-1 functionalized-*N*-aryl-1,2,3,4-tetrahydroisoquinoline derivatives showing biological activities towards cholinergic enzymes. Synthesis was performed using visible-light-promoted photo-redox chemistry, starting from a common intermediate, and the application of this synthetic methodology drastically simplified synthetic routes and purification of desired compounds. All synthesized derivates were divided into four groups based on the substituents in the C-1 position, and their inhibition potencies towards two cholinergic enzymes, acetyl- and butyrylcholinesterase were evaluated. Most potent derivatives were selected, and kinetic analysis was further carried out to obtain insights into the mechanisms of inhibition of these two enzymes. Further validation of the mode of inhibition of cholinergic enzymes by the two most potent THIQ compounds, **3c** and **3i**, was performed using fluorescence-quenching titration studies. Molecular docking studies further confirmed the proposed mechanism of enzymes’ inhibition. In silico predictions of physicochemical properties, pharmacokinetics, drug-likeness, and medicinal chemistry friendliness of the selected most potent derivatives were performed using Swiss ADME tool. This was followed by UPLC-assisted log P determination and in vitro BBB permeability studies performed in order to assess the potential of the synthesized compounds to pass the BBB.

## 1. Introduction

Alzheimer’s disease (AD) is a leading neurodegenerative disorder among elderly individuals, with the prediction that the number of AD patients will more than double in Europe and triple worldwide by 2050, indicating that this disease could become the costliest, deadliest, and the most severe disease of this century [1]. Food and Drug Administration Agency (FDA) approved drugs for AD treatment are either based on the inhibition of acetylcholinesterase (AChE), the enzyme that is part of the cholinergic system or on the inhibition of β-amyloid (Aβ) aggregation [2,3,4]. However, both types of drugs are either not efficient enough and/or have shown significant side effects [1,5,6,7,8]. In this regard, the discovering of a new, safe, and effective drugs for AD treatment and placing them into the medical practice is a highly demanded. 

Currently, the enhancement of cholinergic neurotransmission remains a primary approach in the symptomatic treatment of cognitive and behavioral symptoms associated with mild and moderate stages of AD. This strategy aims to address deficiencies in acetylcholine (ACh) levels, targeting the cholinergic system in order to alleviate cognitive decline and behavioral disturbances faced by individuals at these stages of AD [9]. Two cholinergic enzymes, AChE and butyrylcholinesterase (BuChE), are responsible for breaking down ACh, thus decreasing its level. Although both enzymes can hydrolyze AcCh, AChE is more efficient than BuChE, but the latter plays an important role in regulating AChE activity as well as protecting it from poisoning [10,11]. Moreover, in the late stage of AD, the activity of BuChE is elevated which highlights its importance in maintaining ACh levels and its potential as a target in the treatment of AD. Additionally, both enzymes significantly contribute to enhanced Aβ toxicity. AChE takes part in the pathological formation of highly toxic Aβ−AChE deposits (much more toxic complex than Aβ) by enabling the binding of Aβ to its PAS [12], while BuChE contributes to the transformation of Aβ benign form into its malignant form [11].

Tetrahydroisoquinolines (THIQs) are a privileged class of compounds due to a wide range of medical applications [13,14]. The compounds with this scaffold show anti-inflammatory activity [14], can be ligands for CNS receptors [15] and can possess antitumor activity [16]. Recently published data also show that hybrid compounds with tetrahydroisoquinoline moiety possess strong neuroprotective and anti-Alzheimer effects [17,18,19]. Their anti-Alzheimer effect is mainly attributed to reversible inhibition of AChE and/or BuChE at micromolar concentrations by binding of THIQ motif to PAS sites of both enzymes [20,21,22]. However, THIQ compounds can target not only cholinergic enzymes but also other proteins such as histamine 3 receptor (H3R) [23] and β-amyloid precursor protein cleaving enzyme 1 (BACE1) [18] and reduce levels of toxic Aβ42 [17].

During the time- and resource-consuming process of drug discovery and development, many biologically active molecular structures have been synthesized, but most of them failed to be turned into drugs because of undesirable absorption, distribution, metabolism, excretion, and toxicity (ADMET) profiles. To be able to inhibit synaptic ChEs, new proposed drugs must possess satisfactory lipophilicity and be able to cross the blood−brain barrier. Therefore, in silico or/and experimental prediction of the above-mentioned properties could facilitate and reduce the cost of the process of discoveries of compounds that could represent potential drugs in AD treatment.

The promising anti-Alzheimer potential of tetrahydroisoquinolines motivated us to investigate a new series of *N*-aryl-tetrahydroisoquinoline derivatives as possible inhibitors of cholinergic enzymes. The mechanisms of inhibition of these enzymes by lead compounds are explained by combining kinetic measurements, fluorescent studies, molecular docking, and molecular dynamic studies. Additionally, to assess the pharmacological potential of selected THIQs, their ADME profiles were predicted in silico, while lipophilicity and ability to cross the blood−brain barrier were experimentally evaluated. The obtained results show that designing and searching for new THIQs drugs targeting AChE and BuChE may in the future allow the discovery of therapies that could be potentially more effective in the treatment of neurodegenerative diseases.

## 2. Results and Discussion

### 2.1. Chemistry—Synthesis of Compounds

**Scheme 1:**
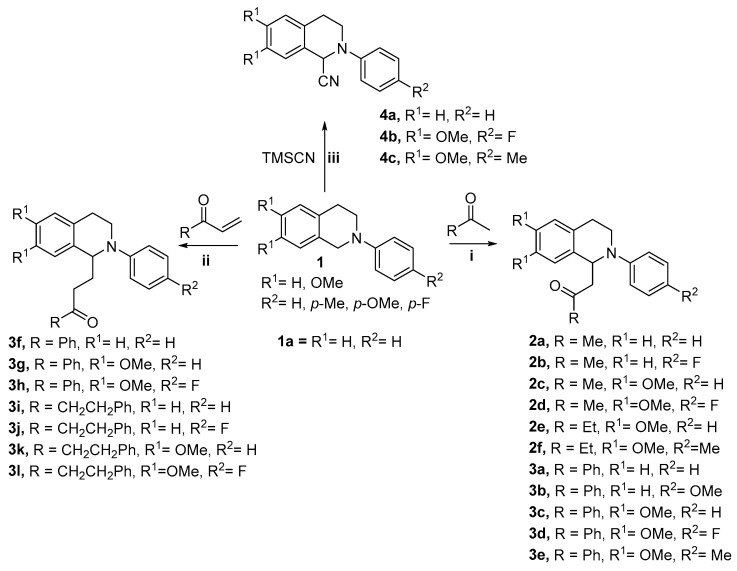
Synthesis of derivatives **2**–**4** *^a^. ^a^ *Reagents and conditions: (i) Ru(bpy)_3_Cl_2_×6H_2_O 1 mol%, L-Proline 30 mol%, ketone 3 equiv., MeOH, r.t. 24 h, 8 W CFL lamp; (ii) Ru(bpy)_3_Cl_2_×6H_2_O 1 mol%, trimethylsilyl cyanide 1.2 equiv., CH_3_CN; r.t. 24 h, 8 W CFL lamp; (iii) Ru(bpy)_3_Cl_2_×6H_2_O 1 mol%, vinyl ketone 4 equiv., TFA 1 equiv., CH_3_CN, r.t. 24 h, 8 W CFL lamp.

The synthetic route for the preparation of desired C1-substituted *N*-aryl-tetrahydroisoquinoline (THIQ) derivatives starting from common intermediate **1** is shown in Scheme 1. All desired compounds were synthesized under the conditions of visible-light-promoted photo-redox catalysis. In a Mannich-type reaction (route **i**), enolizable ketones are used as nucleophiles in the presence of a proline amino catalyst, resulting in the formation of β-amino ketones **2**. The synthesis of γ-amino ketones **3** (route **ii**) was achieved following α-amino radical formation from **1** and its subsequent addition to electrophilic Michael acceptors. In route **iii**, trimethylsilyl cyanide (TMSCN) is used as a nucleophile in a Strecker-type addition to **1**, leading to the formation of α-cyano amines **4**. In total, 22 derivatives with diverse structures and properties were synthesized following the synthetic routes and reactions outlined in Scheme 1. The use of common intermediates and the application of the same synthetic methodology drastically simplified synthetic routes and purification of desired compounds.

### 2.2. Evaluation of the Cholinesterase’ Enzymes Inhibition Potency and SAR Discussion 

**Scheme 2:**
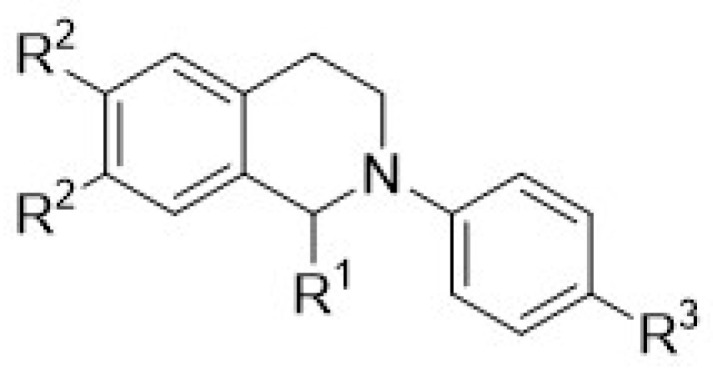
General structure of synthesized compounds.

All synthesized derivates of *N*-aryl-1,2,3,4-tetrahydroisoquinoline were divided into four groups based on the substituents in the R^1^ position (Scheme 2), and their biological profiles were further evaluated, estimating their inhibition potencies towards two cholinergic enzymes, acetyl- and butyrylcholinesterase.

At the outset, the inhibition potency screening tests were performed at a 1 × 10^−5^ M concentration, and the obtained results are summarized in Table 1. R^1^-unsubstituted *N*-phenyl-1,2,3,4-tetrahydroisoquinoline, **1a**, did not show inhibition potential towards both enzymes. On the other hand, the introduction of a substituent at position R^1^ of *N*-aryl-1,2,3,4-tetrahydroisoquinoline leads to an improvement in inhibitory potency against both enzymes, highlighting the significance of this position for THIQ’s inhibition potency, as shown in Table 1. Compounds **2a**–**f** obtained via organocatalyzed Mannich addition of ketones to THIQs were tested first. Compound **2a** containing alkyl-substituted ketone moiety at position R^1^ of THIQ ring showed a low inhibitory activity towards these two enzymes, and an addition of a fluoro substituent at R^3^ (**2b**) led to a double-fold increase in inhibitory activity. Inhibition potencies were observed to be dependent on the chain length of the alkyl-substituted ketone at the R^1^ position. The introduction of one more methylene group into the structure resulted in a decrease in inhibition towards AChE and a loss of inhibition towards BuChE (**2c** vs. **2e**). A better inhibitory activity was obtained by Mannich addition of acetophenone to THIQs, giving compounds **3a**–**e** with a phenyl substituent at the keto group in R^1^ of compounds. These compounds in general showed significantly improved inhibitory activity compared to alkyl-substituted compounds. Compounds **4a**–**c** containing nitrile groups in R^1^ position of *N*-aryl-THIQs showed low inhibition potencies towards cholinesterase enzymes with inhibitory activities lower than 50%. In general, the most promising results were obtained in the **3f**–**l** series of compounds, possessing an additional methylene group between C1 carbon of THIQs and a keto group at R^1^ position.

The screening tests showed that the presence of the methoxy group in R^2^ position of the synthesized compounds led to an increase in the inhibition selectivity towards acetylcholinesterase compared to that towards butyrylcholinesterase, regardless of the substituents in R^1^ position for almost all compounds (Table 1). In contrast to the influence of substituents in R^1^ and R^2^ positions, the role of substituents in R^3^ position was negligible regarding the inhibition potencies of these compounds and independent of substituents’ nature (vide infra). A more detailed inhibition analysis was carried out further with the compounds that showed an inhibition potency higher than 50% in the screening test. These compounds were assayed in the concentration range from 1 × 10^−5^ to 1 × 10^−10^ M towards both enzymes, and the obtained IC_50_ values, as well as the selectivity index (SI), are shown in Table 1. Donepezil and tacrine were used as reference compounds. 

From the results of the target inhibition assays of the selected compounds (Table 1), it was observed that compounds with an R^1^-substituent-possessing phenyl (Ph) group showed low micromolar inhibitory activities against both *ee*AChE and *eq*BChE enzymes. The importance of the Ph group at R^1^ position can be seen from a 2.5-fold increase in inhibitory activity towards AChE of compound **3c** compared to that towards AChE of compound **2c** (similarly for **3a** vs. **2a**; Table 1). The effect of increasing the alkyl chain length between C1 and the keto group from one to two methylene groups was also evaluated. To achieve this, a different synthetic approach was taken; these compounds can be obtained by addition of THIQ α-amino radical to Michael acceptors (reaction path **iii**). In this manner, we synthesized compound **3f**, which showed a double-fold increase in inhibitory activity compared to **3a**. To check the effect of the chain length between the keto and the Ph groups, we synthesized compound **3i**, which showed a further double-fold increase in activity (**3i** vs. **3f**). To summarize, an increase in inhibitory activity towards both enzymes was observed, as the distance of the Ph group from C1 of THIQ increased (**3i** > **3f** > **3a**). The methoxy substitution at R^2^ in β-amino ketone **3c** led not only to an increased activity towards AChE, but also to a decreased activity towards BuChE. In contrast, the methoxy substitution at these positions of γ-amino ketones, **3g** and **3k**, led to three- and four-fold decreases in activity towards AChE, respectively.

The introduction of substituents at R^3^ position led to a decrease of the activity compared to unsubstituted compounds. Fluoro-substituted **3d** had a 2.5-fold lower inhibitory activity towards AChE compared to unsubstituted **3c**. The same behavior was observed for fluoro-substituted **3h** compared to **3g**, for **3j** compared to **3i**, and for **2d** compared to **2c** (Table 1). Similarly, methoxy-substituted **3b** also had a lower inhibition potency towards both enzymes, compared to unsubstituted **3a**; in addition, methyl-substituted **3e** had a lower inhibition potency compared to **3c**. From these results, either electron-withdrawing, fluoro, or electron donating, methoxy or methyl substituents, at R^3^ did not lead to an increase in inhibitory potency.

The structure−activity relationship observed for BuChE was similar to that of AChE, although the activities of the tested compounds were slightly lower compared to their activities towards AChE. The methoxy substitution at R^2^ was found to have a detrimental effect on the activity towards BuChE. 

In general, the structural modifications performed on the compounds were effective, resulting in most derivatives showing a substantial activity. All the compounds in this class exhibited inhibitory rates on AChE below 10 μM.

To summarize, among the studied compounds, **3c** and **3i** exhibited the best inhibitory potency towards AChE, while compound **3i** was also the most potent inhibitor of BuChE among all the tested compounds. This suggests that **3c** could be a promising candidate for further development of AChE-based drugs. On the other hand, **3i** has the potential to be a promising candidate for further development as a dual inhibitor, targeting both AChE and BuChE enzymes and overcoming limitations met only by AChE inhibition.

### 2.3. Kinetic Studies of Enzymes Inhibition

**Figure 1:**
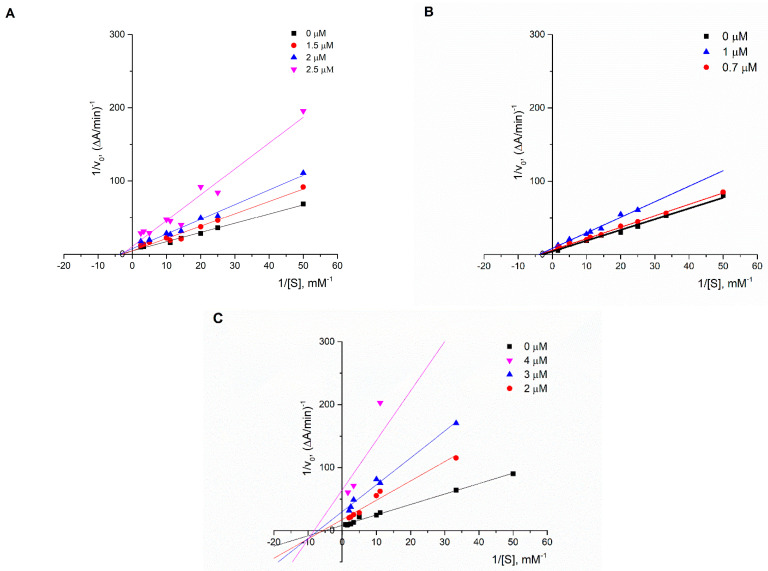
Steady-state inhibition of AChE and BuChE by compounds: (**A**) **3c** and AChE; (**B**) **3i** and AChE; and (**C**) **3i** and BuChE. The Lineweaver-Burk reciprocal plots of the initial velocity in the function of substrate concentrations are presented.

According to the obtained IC_50_ values, **3c** and **3i** compounds were selected as the most potent compounds, and kinetic analysis was further carried out to obtain insights into the mechanisms of inhibition of these two enzymes. Kinetic analysis performed on AChE clearly showed that both compounds inhibited it via the same, non-competitive mode of action (Table 2, Figure 1). However, the mode of inhibition of BuChE for compound **3i** was different from that of AChE. It was shown that BuChE in the presence of **3i** underwent a mixed type of inhibition (non-competitive/uncompetitive) (Table 2, Figure 1). In the case of non-competitive inhibition (AChE), the probability of inhibitor binding to the bare enzyme and the previously formed enzyme−substrate complex is the same, which points out both compounds bound on an allosteric peripheral anionic site (PAS), away from a catalytically active site (CAS). On the other hand, the mixed-type inhibition observed in the case of BuChE indicated that the probability of inhibitor binding to bare and substrate-bound enzymes is not the same. In the case of the non-competitive/uncompetitive mixed-type inhibition, the inhibitor prefers to bind on an allosteric site after formation of an enzyme−substrate complex, thus avoiding formation of choline. The fact that the same compound inhibited two cholinergic enzymes by different modes of inhibition could probably be explained by differences in the structure of PAS sites of AChE and BuChE.

### 2.4. Influence of Inhibitors Binding on the Trp−AChE/BuChE Fluorescence 

**Figure 2:**
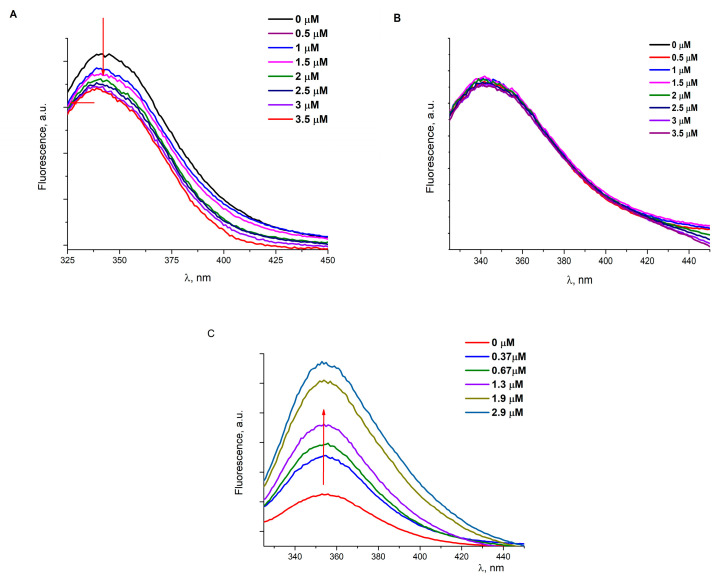
Fluorescence spectra of 5 IU/mL AChE (**A**,**B**) and 5IU/mL BuChE (**C**) in the absence (0 μM) and presence of selected inhibitors: (**A**) compound **3i** and AChE; (**B**) compound **3c** and AChE; (**C**) compound **3i** and BuChE.

In order to validate the mode of inhibition of cholinergic enzymes by the two most potent THIQ compounds, **3c** and **3i**, fluorescence-quenching titration studies were performed. From the kinetic measurement, we concluded that compounds **3c** and **3i** inhibited AChE by a pure non-competitive mode of action. This means that both compounds were bound to the allosteric site of the enzyme, away from the ACh-binding site, and that binding affinity is same for both the enzyme and the enzyme−substrate complex. According to the literature data, THIQ-based compounds achieve their inhibitor potencies towards both enzymes via π-π interaction of benzene’s ring of THIQ fragment with Trp residues in the PAS site [20]. With this in mind, it is reasonable to anticipate that the PAS site could also serve as an allosteric site in this case. Consequently, the binding of the above-mentioned compounds, **3c** and **3i**, may influence the Trp fluorescence of AChE. This assumption was confirmed for compound **3i**, as in this instance, the quenching of Trp fluorescence was observed (Figure 2A). However, in the case of compound **3c**, there was no quenching of Trp fluorescence. 

Compound **3i** induced quenching of Trp fluorescence with bimolecular quenching constants, k_q_, higher than ones for the diffusion-controlled process, indicating that Trp residues could be involved in the binding interaction. The quenching study also pointed out that there was one binding site on the enzyme (Table 3). The observed bathochromic shift indicates that the binding of compound **3i** induced conformational changes in AChE, resulting in Trp residues being surrounded by a more hydrophobic environment (Figure 2A). The absence of Trp fluorescence quenching observed in the case of compound **3c** could be attributed to the compound’s inability to quench Trp residues, as confirmed by the recorded UV/Vis spectra, which showed the absence of interaction between **3c** and Trp. This fact also pointed out Trp residues might not be involved in binding. Additionally, the fluorescence-quenching titration studies performed on the Trp solution also confirmed the inability of **3c** for Trp quenching (unpublished results). However, it does not rule out the possibility of the compound’s binding at the PAS, which was in fact confirmed with docking studies (vide infra). Contrary to this, the appearance of hypochromic shifts was observed for compound **3i** in the UV/Vis spectrum (unpublished results), pointing out the possibility of an interaction between Trp residues and compound **3i**.

On the contrary to the mechanism of AChE inhibition, the mechanism of BuChE inhibition by **3i** is a mixed type of noncompetitive inhibition (noncompetitive–uncompetitive). As in the previous case, **3i** was also bound on the allosteric site, but here, it rather preferred to bind on an enzyme−substrate complex. The effect of compound **3i** on the Trp fluorescence of BuChE was also different. In this case, the binding of **3i** caused such conformational changes of BuChE that Trp residues became more exposed to the solvent. Therefore, previously quenched Trp fluorescence was suppressed, and the fluorescence intensity of BuChE was increased as a consequence of this conformational change [24]. Usually, the interaction between the indole ring of Trp and ionizable side chains of His or Arg in its neighborhood could lead to quenching of Trp fluorescence [25], and **3i** appears to break these interactions, thus causing an increase in the fluorescence intensity. The observed differences in the influence of **3i** on Trp fluorescence of AChE and BuChE could be explained by the three key aromatic residues present in the PAS of AChE (Tyr72, Tyr341, and Trp 286), which are absent in BuChE [26]. Exactly, the absence of these residues in the PAS site of BuChE contributes to the transient binding of BuChE inhibitors to its PAS site and their subsequent sliding down the gorge to interact with the other part of the binding site. This mode of binding is in accordance with mixed-type inhibition observed in the kinetic study [26]. 

### 2.5. Docking Studies

**Figure 3:**
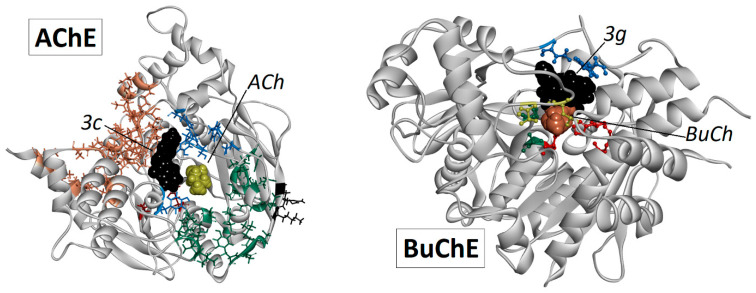
Binding sites of **3c** (**left**) and **3i** (**right**) derivatives (black colored), acetylcholine (Ach; yellow colored), and butyrylcholine (BuCh; orange colored) to AChE and BuChE.

**Figure 4:**
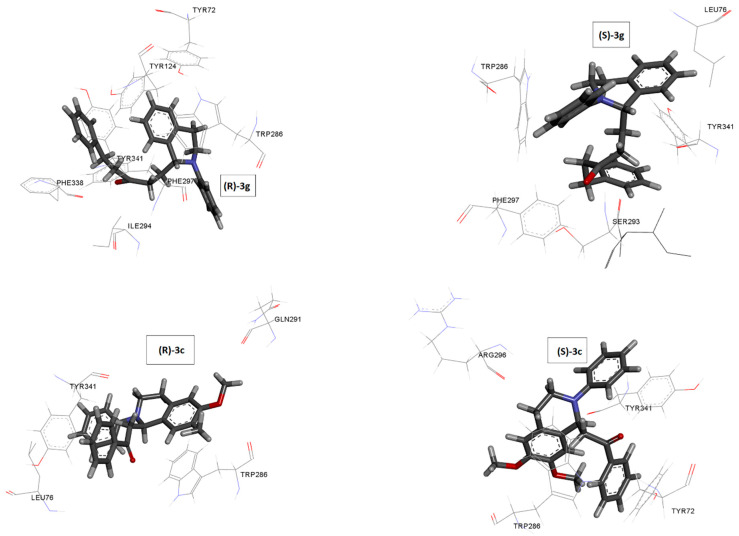
The amino acid environment of the investigated THIQs, **3i** (**top**) and **3c** (**bottom**), in their binding sites on AChE.

To gain insights into the mechanism of enzymes’ inhibition by selected THIQs at the molecular level, docking studies were performed using both stereoisomers of THIQs. The results of the docking study performed on AChE (pdb code: 1C2B) pointed out **3c** and **3i** had the same binding site (Figure 3), which was located between *Tyr72* and *Tyr341* amino acid residues from the peripheral anion site (PAS) and *Gln291*, *Ser293*, *Arg296*, *Phe297* residues. The π-π interactions (stacking and T-shaped interactions) were mainly responsible for binding, while the contribution of hydrogen binding was significantly smaller. The illustration of the amino acid environment of the investigated THIQs in the binding site on AChE is presented in Figure 4. Phenyl groups from THIQ ring and from R1 chain contributed to binding of **3i** on AChE through their π-π interactions with *Tyr72* as well as with *Tyr341* and *Tyr124* separately. On the contrary to **3i**, for **3c**, the involvement of an *N*-aryl group into π-π interactions was observed instead of contribution of phenyl from R1 chain, indicating the importance of this group for BuChE inhibition.

Quantitative results of the docking studies are shown in more detail in Table 4. The estimated binding energies were in the range from −7.5 to −9.9 kcal/mol, while R stereoisomer binding was slightly stronger. The mentioned THIQs had a significantly higher binding affinity to AChE (9.1 kcal/mol) than acetylcholine, which was bound in the active site (to the catalytic triad), with a binding energy of −4.2 kcal/mol. 

**Figure 5:**
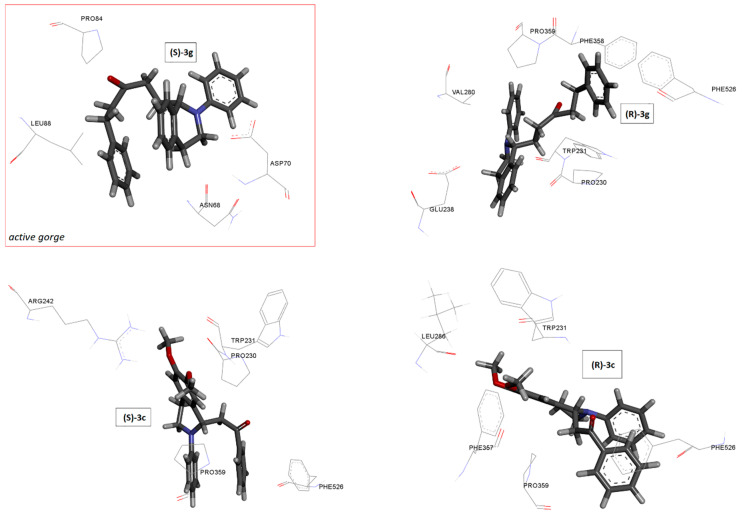
The amino acid environment of the investigated THIQs (**3i** (**top**) and **3c** (**bottom**)) in the binding site on BuChE.

To explain the mechanism of inhibition of BuChE by **3i**, a molecular docking study was also performed using BuChE structures relaxed for 200 ns by molecular dynamics. The obtained results of the docking study have shown that S-stereoisomer of **3i** was bound in the active gorge (Figure 5), between catalytic triad (CAS: *Ser198*, *His438*, and *Glu325*), the choline-binding site (CBS: *Trp82* and *Tyr332*), and the peripheral anionic site *(*PAS: *Asp70* and *Tyr332*) with similar binding energy for both stereoisomers (Table 4). Van der Waals interactions mainly contributed to binding, and S stereoisomer was slightly stronger bound compared to R isomer (Figure 5) as in the case of AChE (Table 4). 

### 2.6. ADME Prediction, log P, and the Blood−Brain Barrier Permeability Determination 

During the discovery and development of the drug, in addition to its biological activity, the proposed compounds must have an appropriate molecular weight, a polar surface area, a limited number of donor and acceptor H atoms, and appropriate lipophilicity and solubility. In addition, another criterion must be met in the treatment of AD—the compounds must cross the blood−brain barrier. A first glance into the drug-likeness of a test compound can be obtained using various in silico methods, but these predictions must be proven by experimental methods before they are accepted or rejected. To assess whether the biologically active synthesized THIQs meet the criteria to be considered drugs, in silico predictions were initially made after which the obtained results were confirmed for the most potent compounds.

#### 2.6.1. In Silico ADME Predictions for Selected THIQs 

In silico predictions of physicochemical properties, pharmacokinetics, drug-likeness, and medicinal chemistry friendliness of selected THIQs were performed using the free access Swiss ADME tool [27,28]. This method gives the first assessment, if selected THIQs could be considered as drugs in AD treatment. The obtained results show that all investigated THIQs could pass the BBB while Lipinski rule 5 was not satisfied only for **3f**, **3i**, and **3j** based on the predicted MlogP for these compounds (MlogP > 4.15; MlogP—an archetype of topological method relying on a linear relationship with 13 molecular descriptors’ values (Table 5) [29]. In this regard, it is possible to conclude that all synthesized compounds, except these ones, could be potential candidates for drugs in AD disease. 

**Figure 6:**
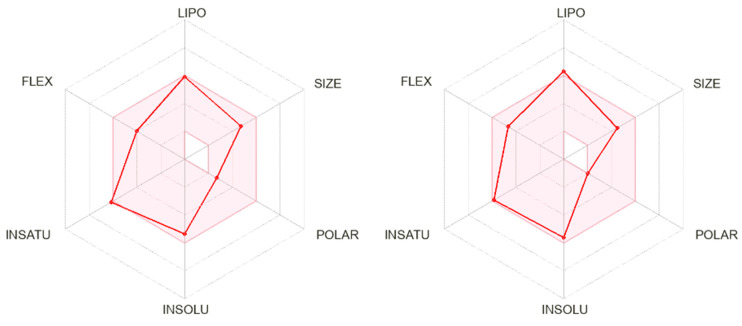
Bioavailability radar images obtained for compounds **3c** (**left**) and **3i** (**right**) from Swiss ADME. The pink area represents the optimal range for each property (lipophilicity: XLOGP3 between −0.7 and +5.0; size: MW between 150 and 500 g/mol; polarity: TPSA between 20 and 130 Å2; solubility: log S not higher than 6; saturation: fraction of carbons in the sp^3^ hybridization not less than 0.25; flexibility: no more than 9 rotatable bonds).

To clearly emphasize the properties of the most potent compounds, **3c** and **3i,** the bioavailability radars are shown in Figure 6. The optimal value range for each property is represented by the pink area. For compound **3c**, oral bioavailability was predicted, which was not the case for compound **3i** due to the higher logP value.

#### 2.6.2. Ultra-High-Performance Chromatography log P Determination and the In Vitro BBB Permeability Study

As mentioned above, one compound could be considered as a potential drug in the treatment of Alzheimer’s disease, if it meets two important requirements, appropriate values of lipophilicity and ability to pass the BBB. Lipophilicity is one of the physicochemical properties of the drug that has influence on drug pharmacokinetics, impacting on drug uptake and metabolism and influencing the promotion of off-target binding for increasing the drug’s toxicity [30]. According to SwissADME, all selected THIQ compounds satisfied Lipinski rules 5 except **3f**, **3i**, and **3j** due to higher logP values. However, since **3i** is also one of the most potent compounds next to **3c**, logP values were also experimentally determined using the UPLC method (Table 6). Experimentally determined logP values for compounds **3c**, **3f**, and **3i** are in the range convenient for drugs in neurodegenerative disease according to “Lipinski rules 5” (Table 6). These findings hint the possibility of passing the blood−brain barrier, but they did not prove it. 

To evaluate the potential of the synthesized compounds to penetrate the BBB, a parallel artificial membrane permeation assay for the BBB (PAMPA-BBB) was carried out [31]. This model can identify compounds as either BBB permeable (BBB+) or non-permeable (BBB−) with reasonable accuracy. Based on the obtained permeability (Pe), compounds could be divided into five groups: non-permeable compounds with P_e_ < 7.24 ×10^−7^, low permeable compounds with 7.24 ×10^−7^ < P_e_ < 2.19 ×10^−6^, medium permeable compounds with 2.19 ×10^−6^ < P_e_ < 4.68 ×10^−6^, and high permeable compounds with logP_e_ > 4.68 ×10^−6^. We conducted a PAMPA assay for the most potent compounds, **3c** and **3i**, and for **3f**, using verapamil and caffeine as positive and negative controls, respectively. The obtained results are also presented in Table 6. While all compounds exhibited logP values suitable for drugs in the treatment of Alzheimer’s disease, only **3c** successfully crossed the artificial blood−brain barrier. The remaining compounds, **3i** and **3f**, despite having similar logP values, possessed excessively high lipophilicity, making them impossible to be detected. Based on the summary of the obtained results, compound **3c** was chosen as the lead compound that possesses promising properties for further evolution. Further synthetic modifications of **3i** might help in obtaining compounds with improved and desired properties.

**Table 6:** Determined permeabilities and lipophilicities of selected THIQ compounds.

Compound	logP	PAMPA-BBB Permeability P_e_; 10^−6^ cm s^−1^
**3c**	3.39	47.5
**3f**	3.66	High hydrophobicity; it retained in the membrane
**3i**	3.78	High hydrophobicity; it retained in the membrane
Verapamil × HCl	3.80 [32]	15.8
Caffeine	−0.07 [33]	1.47

## 3. Conclusions

In summary, our study revealed new *N*-aryl-1,2,3,4-tetrahydroisoquinoline-based derivatives as promising candidates for further development as the potential drugs in the treatment of Alzheimer’s disease. To the best of our knowledge, this is the first time that R^1^ position in the THIQ moiety is highlighted as the leading position, responsible for biological activity towards cholinergic enzymes. Furthermore, the presence of the methoxy group in R^2^ position led to an increase in the inhibition selectivity towards acetylcholinesterase compared to that towards butyrylcholinesterase. In contrast to influence of substituents in R^1^ and R^2^ positions, the role of substituents in R^3^ position was negligible regarding the inhibition potencies of these compounds and independent of substituents’ nature.

Alkyl or nitrile groups in R^1^ position of *N*-aryl-THIQs (**2** and **4** series of compounds) have not shown significant improvement of inhibition potency, showing moderate cholinesterase inhibitory activity. On the other hand, the compounds with a phenyl group in position R^1^, connected to C1 of THIQ through linkers of various lengths (**3a**, **3c**, **3f**, and **3i**) represent the most potent compounds with the inhibition potency at low micromolar concentration towards AChE or both enzymes. According to docking and molecular dynamic studies performed on the most potent compounds, they bound to PAS. This finding is supported by mechanistic evaluation, i.e., kinetic and fluorescence measurements. This is very significant, since binding to PAS opens the possibility of avoiding AChE-assisted formation of amyloid plaques by blocking this site for interaction with amyloid. The most important interactions that contributed to binding of THIQ to PAS were π-π interactions. All the selected most potent inhibitors possessed appropriate logP values, but according to the PAMPA test, only compound **3c** was able to cross the BBB. Therefore, **3c** was selected as the lead compound that acted as an AChE inhibitor binding to the peripheric active site of this enzyme and that fulfilled one of the most important requirements in discovery of drugs for AD—it crosses the BBB. 

## 4. Experimental Section

### 4.1. Chemicals

Acetylcholinesterase (AChE) from electric eel (C3389), butyrylcholinesterase (BuChE) from horse serum (C1057), acetylthiocholine iodide (ASChI), S-butyrylthiocholine chloride (BSChCl), 5,5′-dithio-bis(2-nitrobenzoic acid) (DTNB), dimethyl sulfoxide (DMSO), dodecane, (NH_4_)_2_HPO_4_, Na_2_HPO_4_, NH_4_H_2_PO_4_, KH_2_PO_4_, Tris, verapamil, and caffeine were purchased from Sigma-Aldrich, Steinheim am Albuch, Germany. Porcine brain lipid (PBL) was obtained from Avanti Polar Lipids, Darmstadt, Germany.

Stock solutions (10 mM) of synthesized derivates of tetrahydroisoquinoline compounds were prepared daily by solving solid compounds in DMSO or in the acetonitrile when UPLC measurements were performed. 

The chemical reactions were monitored by thin-layer chromatography using Merck 60 F254 precoated silica gel plates (0.25 mm thickness). Preparative thin-layer chromatography was performed using Merck 60 F254 silica gel purchased from Merck KGA, Germany. Column chromatography was carried out on silica gel (12–26, ICN Biomedicals, Costa Mesa, CA, USA) using petrol ether/ethyl acetate as eluents. 1H-NMR and 13C-NMR spectra were measured on, a Bruker Ultrashield Advance III, Germany, spectrometer (^1^H at 500 MHz, ^13^C at 125 MHz) and Varian 400 sprectrometer, USA (^1^H at 400 MHz, ^13^C at 100 MHz) using CDCl_3_ as a solvent with TMS as an internal standard. Chemical shifts (δ) were given in parts per million (ppm), and coupling constants were given in Hertz (Hz). The proton spectra were reported as follows δ/ppm (multiplicity, number of protons, and coupling constant *J*/Hz). High-resolution mass spectrometry (HRMS) spectra were recorded only for new compounds using an Orbitrap Exploris 240 mass spectrometer (Thermo Fisher Scientific, Waltham, MA, USA), with heated electrospray ionization (HESI) as an ion source. IR spectra were measured on a PerkineElmer FT-IR 1725X, Waltham, MA, USA spectrophotometer using the ATR technique. The peak intensities were defined as very strong (vs), strong (s), middle (m), or weak (w). Check SI for details.

### 4.2. In Vitro Inhibitory Evaluation on AChE/BChE

The abilities of synthesized 22 compounds to inhibit AChE and BuChE activity were determined following Ellman’s procedure using a Perkin Elmer Lambda 35 spectrophotometer with thermostated 1.00 cm quartz cells [34]. AChE’s and BuChE’s stock solutions were diluted with 20 mM TRIS to give 1 unit per mL of enzymes’ activity. ASChI and BSChCI solutions (0.075 M) were prepared in deionized water. A 0.01 M DTNB solution was prepared with 0.1 M phosphate buffer (pH 7) containing 0.15% (*w*/*v*) sodium bicarbonate. A 0.1 M phosphate buffer (pH 8) was used for assay. pH was adjusted to 8.0 ± 0.1 by adding aliquots of concentrated HCl. Stock solutions (10 mM) of investigated compounds were prepared daily by dissolving solid compounds in DMSO. Diluted solutions were also prepared in DMSO. 

Briefly, enzymes’ assays were prepared as follows. An appropriate volume of 0.1 M phosphate buffer (pH 8.0), 286 μM DTNB, the test compound in a desired concentration (1 × 10^−5^ M for the screening test) or in the increasing concentrations (for IC_50_ determination), and 0.0285 UI/mL AChE (BuChE) were preincubated at 37 °C for 6 min. Thereafter, 20 μL of ASChI or BSChCI was added, after which the reaction was initiated. After incubation at 37 °C for 6 min, the absorption was measured spectrophotometrically at 412 nm. For the blank value, the addition of 20 μL of buffer replaced the enzyme solution while an addition of 7 μL of DMSO replaced the inhibitor solution. The control solution contained 7 μL of DMSO instead of the inhibitor. The screening test was performed at a 1 × 10^−5^ M concentration, and the compounds with more than 50% of inhibition were further assayed using dilution series of up to eight increasing concentrations (10^−5^ M to 10^−9^ M). The inhibition potency was presented as the dependence on the relative enzyme activity (REA) as the function of the compound’s concentration. IC_50_ values were determined graphically from Hill curves using Origin 9 Microsoft Windows. 

### 4.3. Kinetics Studies of ChE’s Inhibition

Kinetic studies of ChE enzymes were also performed using the above-mentioned modified Ellman’s method [34]. The preincubation time was 6 min, while the incubation time was 2 min. The type of enzyme inhibition was justified by the Lineweaver–Burk plot (double reciprocal plot) for selected inhibitor concentrations, and substrate concentrations ranged from 0.01 to 1 mM. The kinetic parameters K_m_, V_max_, and K_i_ were determined. Graphs were plotted using Origin9 for Microsoft Windows.

### 4.4. Fluorescence Measurements

Fluorescence titration studies were conducted using the Agilent Cary Eclipse fluorescence spectrophotometer. Fluorescence-quenching spectra were recorded within the wavelength range of 320–450 nm, with an excitation at 295 nm at room temperature. Excitation and emission slit widths were both set at 5 nm, and the scanning rate was fixed at 9600 nm/min. The concentration of enzymes was 5 IU/mL. The incubation time between each addition of THIQ aliquots was 6 min. 

### 4.5. In Silico Prediction of ADME

In silico predictions of ADME properties for selected THIQs were performed using the free access Swiss ADME tool [27,28]. 

### 4.6. Determination of Partition Coefficient (logP) of Tested Compounds by UPLC Methods

The partition coefficient logP values of the most potent compounds were determined using a Waters ACQUITY UPLC system equipped with a tunable UV detector (from 200 to 500 nm) controlled by the Empower 3 software. The column used for logP determinations was ACQUITY UPLC BEH C18 column with the dimensions 1.7 μm × 100 mm × 2.1 mm (Waters). The measurements were performed in the isocratic mode. The eluent flow rate was 0.25 mL min^−1^, and the injection volume was 5 μL. The mobile phase composition was a different percentage of acetonitrile and H_2_O (we ran a chromatogram for each compound with different eluent compositions and the acetonitrile fraction ranging from 64% to 91%). The wavelength of the optical detection was 256 nm, the retention times for every examined compound under certain experimental conditions were determined. At the beginning, the compound’s retention factor, k’, was determined for different percentages of acetonitrile in the mobile phases. The column’s dead time was determined by the injection of sodium bromide. After that, the retention factors for a pure aqueous eluent (log k_w_) were determined for the compounds with previously known logP_o/w_ values as well as for the investigated compounds. Thereafter, obtained *logP* values were correlated with their previously known *logP* values, and the following equation was obtained (1):

(1) $$logP=1.065+0.770logK_{w}$$

where *logK_w_* is the retention factors for a pure aqueous eluent of the investigated compound [35]. 

### 4.7. PAMPA-BBB Permeability Assay

Parallel artificial membrane permeability assay (PAMPA) as an in vitro blood−brain barrier (BBB) test was performed to evaluate the abilities of the most potent compounds penetrating the brain. The assay was performed according to the method reported by Di et al. with slight modification [31]. Commercial drugs, verapamil, and caffeine were used to validate the protocol. Determinations were carried out on a MultiScreen-IP PAMPA assay from Millipore Corporation (Burlington, MA, USA) using porcine polar brain lipids as the lipid barrier and calculating the permeability, P_e_, for each compound [36]. The concentrations of compounds in donor and acceptor wells were quantified by UPLC with a UV/DAD detector at λ_max_ of 258 nm for investigated compounds and at 272 nm and 278 nm for caffeine and verapamil, respectively. Each sample was analyzed with at least three independent experiments in three wells. The obtained permeability (P_e_) values of verapamil and caffeine are in good accordance with the previously published literature data, confirming the validity of the performed assay [37]. 

### 4.8. Quantum-Mechanical Calculations

To explain the observed differences in the inhibitor activities of investigated 1,2,3,4-tetrahydroisoquinolines (THIQs), in the first step of the theoretical study, the structures of **3c** and **3i** were optimized by Gaussian09 software [38] using the ωB97X-D method and the 6-311++g** basis set. The solvation energies for ligands were also taken into account, which was calculated by SMD method (the dielectric constant (ε) for water is 78.39), using the ωB97XD functional and the 6-311++g** basis set.

### 4.9. Molecular Docking

Preparation of enzymes for docking studies: Molecular docking studies were performed to investigate the binding modes of investigated THIQs and the potential mechanism of inhibition of acetylcholinesterase (AChE) and butyrylcholinesterase (BuChE). As a model system for acetylcholinesterase, the protein extracted from the crystal structure with PDB code 1C2B was chosen [39]. The model system for butyrylcholinesterase was also extracted from Protein Data Bank, from the structure of the protein with PDB code 1P0P [40]. The structures of the isolated enzymes were obtained by removing water molecules and other ligands, crystallized together with the enzyme. The preparation of enzymes and the optimized THIQs (**3c** and **3i**) was conducted in the AutoDockTools 1.5.6. program.

Docking in AutoDock Vina: The AutoDock Vina 1.2.3. software [41] was used to dock the investigated THIQs onto the whole BuChE and AChE, to determine the probable binding sites of the inhibitor. A docking study was performed on whole enzymes to determine whether ligands had a higher affinity for other sites relative to the active site. Cleaned enzyme structures and optimized structures of 1,2,3,4-tetrahydroisoquinolines were used for docking studies. During the docking calculations, the enzyme structure was kept rigid, while all single bonds of THIQs were set to be rotational. In each case, 100 runs (100 conformations of the enzyme/ligand system) were produced and clustered by similarity. For visualization of target−ligand interactions during docking studies, the BIOVIA Discovery Studio was used [42].

To obtain the relaxed structure of BuChE, the molecular dynamics simulation was performed with WebGro [43], a fully automated online tool, using GROMOS96 43a1 forcefield [44]. The starting structure of BuChE (PDB code: 1P0P) was solvated in a cubic box of water, followed by neutralization and addition of NaCl (the total concentration of salt was 150 mM). SPC was selected as a solvent model. To remove close contacts between atoms, the structure of the enzyme was minimized. The steepest descent algorithm (5000 steps) was applied for this purpose. The MD simulations were performed at a constant temperature (300 K) and a fixed pressure (1.0 bar), with an approximate number of frames per simulation of 1000, while the simulation time was set to 100 ns. The trajectory was analyzed and visualized with the program VMD (version 1.9.2) [45], and the obtained structure from the trajectory (in open form of AChE) was further used for molecular docking studies.

## Figures and Tables

**Table 1:** The inhibition potencies of the synthesized compounds and selectivity indexes towards *ee*AChE and *eq*BuChE. The preincubation time was 6 min, the incubation time was 6 min, and the temperature was 37 °C.

Compound	Structure	Residual Activity at 1 × 10^−5^ M		IC_50_, μM		Selectivity Index for AChE ^a^
		AChE	BuChE	AChE	BuChE	
**1a**	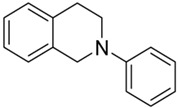	n.a.	11	>10	>10	-
**2a**	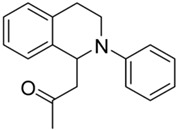	30	43	>10	>10	-
**2b**	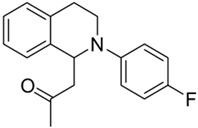	64	47	5.46 ± 0.58	8.90 ± 2.03	1.63
**2c**	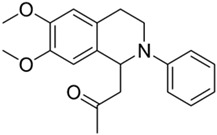	68	2	5.04 ± 0.28	>10	-
**2d**	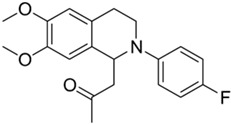	12	15	>10	>10	-
**2e**	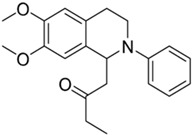	20	n.a.	>10	n.d.	-
**2f**	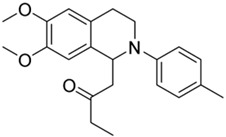	n.a.	n.a.	n.d.	n.d.	-
**3a**	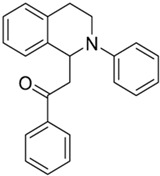	100	54	4.29 ± 0.09	8.26 ± 1.05	1.93
**3b**	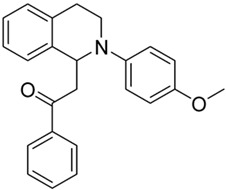	59	20	9.05 ± 0.81	>10	-
**3c**	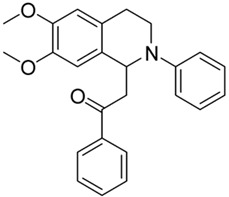	100	37	1.95 ± 0.23	>10	>5
**3d**	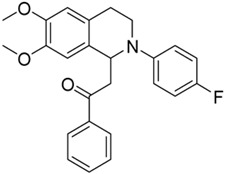	70	20	4.89 ± 0.15	>10	-
**3e**	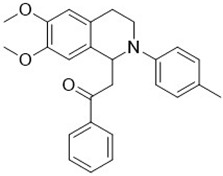	45	n.a.	>10	n.d.	-
**3f**	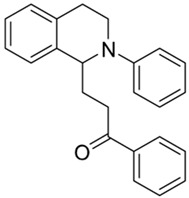	100	75	2.42 ± 0.09	6.28 ± 0.36	2.59
**3g**	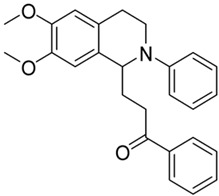	75	30	6.78 ± 0.22	8.01 ± 0.55	1.18
**3h**	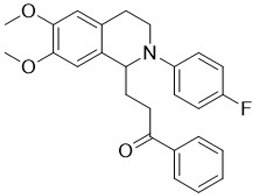	40	10	>10	>10	-
**3i**	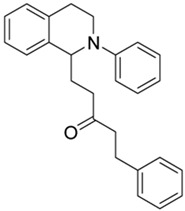	100	100	1.11 ± 0.04	2.72 ± 0.20	2.45
**3j**	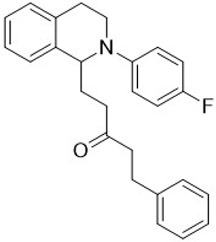	100	100	2.91 ± 0.09	4.52 ± 0.15	1.55
**3k**	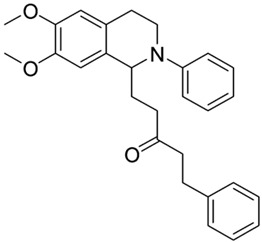	70	30	4.02 ± 0.19	>10	-
**3l**	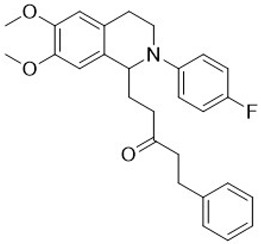	45	5	>10	>10	-
**4a**	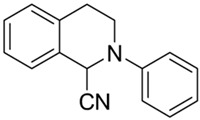	12	23	>10	>10	-
**4b**	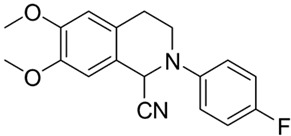	20	20	>10	>10	-
**4c**	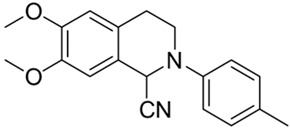	18	30	>10	>10	-
**Tacrine**	-	-	-	0.06 ± 0.001	0.008 ± 0.001	0.13
**Donepezil**	-	-	-	0.02 ± 0.001	3.67 ± 0.05	183

All tests were performed in three independent experiments to give data as mean ± SD. Tacrine (*ee*AChE, IC_50_ = 0.06 μM, *eq*BChE, and IC_50_ = 0.008 μM) and donepezil (*ee*AChE, IC_50_ = 0.02 μM, *eq*BuChE, and IC_50_ = 3.67 μM) were engaged as a positive control. n.a.—no activity; n.d.—not determined; the inhibitory activity was less than 50% at a concentration of 10 μM. ^a^ The selectivity index for AChE was defined as IC_50_(BChE)/IC_50_(AChE).

**Table 2:** IC_50_ and K_i_ values obtained for inhibition of AChE and BuChE for compounds **3c** and **3i**.

Enzyme	Inhibitor	IC_50_, μM	K_i_, μM	Type of Inhibition
AChE	**3c**	1.91 ± 0.11	1.78 ± 0.10	Non-competitive
	**3i**	1.11 ± 0.04	1.06 ± 0.01	Non-competitive
BuChE	**3c**	n.a.	/	/
	**3i**	2.71 ± 0.20	1.04 ± 0.1	Mix non-competitive/uncompetitive

n.a.—no activity.

**Table 3:** Quenching and binding parameters for the **3i**−AChE interaction under experimental conditions.

	Ksv, M^−1^	k_q_, M^−1^s^−1^	K_a_, M	n
**3i**	(6.96 ± 0.05) × 10^4^	(6.96 ± 0.05) × 10^14^	(3.31 ± 0.05) × 10^4^	0.94 ± 0.04

**Table 4:** Configurations (Conf), binding energies (ΔE, in kcal/mol), and numbers of binding sites (No) of investigated THIQs derivatives, **3i** and **3c**, on AChE and BuChE.

Label of Compounds	AChE			BuChE		
	Conf	ΔE	No	Conf	ΔE	No
**3c**	R	−9.2	1	R	−7.9	/
	S	−8.9	1	S	−7.5	/
**3i**	R	−9.9	1	R	−8.7	1
	S	−9.2	1	S	−8.2	1

**Table 5:** Predicted parameters (ADME test) for biologically active THIQ compounds using SwissADME.

	Compounds												
Parameters	2b	2c	3a	3b	3c	3d	3f *	3g	3i *	3j *	3k	Tacrine	Donepezil
Mw, g/mol	283.3	325.4	327.4	357.4	387.5	405.5	341.5	401.5	369.5	387.5	429.6	198.3	379.5
TPSA, Å^2^	20.3	38.8	20.3	29.5	38.8	38.8	20.3	38.8	20.3	20.3	38.8	38.9	38.8
ClogP	3.45	3.09	4.25	4.21	4.16	4.48	4.57	4.46	5.00	5.26	4.90	2.59	4.00
ilogP	2.58	2.89	3.09	3.29	3.38	3.50	3.36	3.51	3.76	3.61	3.99	2.09	3.92
MlogP	3.58	2.46	4.08	3.68	3.31	3.68	4.30	3.51	4.71	5.08	3.91	2.33	3.06
XlogP	3.36	3.21	4.92	4.89	4.86	4.96	5.28	5.22	5.51	5.61	5.45	2.71	4.28
WlogP	3.62	3.08	4.36	4.37	4.38	4.93	4.75	4.77	5.07	5.63	5.08	2.70	3.83
logS (ESOL)	−3.94	−3.92	−5.24	−5.30	−5.37	−5.53	−5.47	−5.60	−5.62	−5.77	−5.76	−3.27	−4.81
No. H-bond acceptor	2	3	1	2	3	4	1	3	1	2	3	1	4
No. H-bond donor	0	0	0	0	0	0	0	0	0	0	0	1	0
No. rotable bonds	3	5	4	5	6	6	5	7	7	7	9	0	6
BBB permeation	yes	yes	yes	yes	yes	yes	yes	yes	yes	yes	yes	yes	yes
LogK_P_ (skin permeation), cm/s	−5.64	−6.01	−4.80	−5.01	−5.21	−5.25	−4.63	−5.04	−4.64	−4.68	−5.05	−5.59	−5.58
Lipinski’s rule violations	no	no	no	no	no	no	yes	no	yes	yes	no	no	no
Bioavailability score	0.55	0.55	0.55	0.55	0.55	0.55	0.55	0.55	0.55	0.55	0.55	0.55	0.55
GI absorbtion	high	high	high	high	high	high	high	high	high	high	high	high	high
PAINS alerts	0	0	0	1	0	0	0	0	0	0	0	0	0
P-pg substrate	yes	yes	yes	yes	yes	yes	yes	yes	yes	yes	yes	yes	yes

* MlogP > 4.15.

## Data Availability

Data are contained within the article and Supplementary Materials.

## Supplementary Materials

The following supporting information can be downloaded at: https://www.mdpi.com/article/10.3390/ijms25021033/s1.
