# Supplementary material for: Targeting Alzheimer’s Disease: Evaluating the Efficacy of C-1 Functionalized N-Aryl-Tetrahydroisoquinolines as Cholinergic Enzyme Inhibitors and Promising Therapeutic Candidates
Source: Int J Mol Sci. 2024 Jan 14;25(2):1033. doi: 10.3390/ijms25021033 (PMC10816625; doi:10.3390/ijms25021033)
Supplement: Supplementary file 1 [file ijms-25-01033-s001.zip › ijms-2815775-supplementary.pdf]

Supplementary Material

Targeting Alzheimer's Disease: Evaluating the Efficacy of C-1 Functionalized N-Aryl-Tetrahydroisoquinolines as Cholinergic Enzyme Inhibitors and Promising Therapeutic Candidates

Dunja Jovanović [1], Ana Filipović [2], Goran Janjić [2], Tamara Lazarević-Pašti [1], Zdravko Džambaski [2], Bojan P. Bondžić [2,*] and Aleksandra M. Bondžić [1,*]

[1] Vinča Institute of Nuclear Sciences, National Institute of the Republic of Serbia, University of Belgrade,
P.O. Box 522, 11000 Belgrade, Serbia; dunja.jovanovic@vin.bg.ac.rs (D.J.); tamara@vin.bg.ac.rs (T.L.-P.)

[2] Institute of Chemistry, Technology and Metallurgy, National Institute of the Republic of Serbia, University of Belgrade, Njegoševa 12, 11000 Belgrade, Serbia; ana.filipovic@nanosys.ihtm.bg.ac.rs (A.F.); goran.janjic@ihtm.bg.ac.rs (G.J.); zdravko.dzambaski@ihtm.bg.ac.rs (Z.D.)

* Correspondence: bojan.bondzic@ihtm.bg.ac.rs (B.P.B.); aleksandrab@vin.bg.ac.rs (A.M.B.)

General Remarks:

All reactions were monitored by thin-layer chromatography using Merck 60 F254 precoated silica gel plates (0.25 mm thickness). Preparative thin layer chromatography was performed using Merck 60 F254 silica gel purchased from Merck KGA. Column chromatography was carried out on silica gel (12-26, ICN Biomedicals) using petrol ether/ethyl acetate as eluents. 1H-NMR and 13C-NMR spectra were measured on a Bruker Ultrashield Advance III spectrometer (^{1}H at 500 MHz, ^{13}C at 125 MHz) and Varian 400 sprectrometer (^{1}H at 400 MHz, ^{13}C at 100 MHz) using $CDCl_3$ as solvent with TMS as internal standard. Chemical shifts (δ) are given in parts per million (ppm) and coupling constants are given in Hertz (Hz). The proton spectra are reported as follows δ/ppm (multiplicity, coupling constant J/Hz, number of protons). High-resolution mass spectral analyses (HRMS) were carried out using Bruker ESI-TOF MS. IR spectra were measured on a PerkineElmer FT-IR 1725X spectrophotometer using ATR technique. The peak intensities are defined as very strong (vs), strong (s), middle (m) or weak (w). Melting points were determined on a Stuart SMP10 apparatus.

General procedure I, THIQ oxidation/Mannich reaction

Tetrahydroisoquinoline (0.25 mmol, 1 equiv.), $Ru(bpy)_3Cl_2*6H_2O$ (0.0025 mmol, 1 mol%), L-Proline (0.0025 mmol, 0.1 equiv.) and ketone (5-10 equiv.) were added to CH_3CN (1 mL) and stirred at room temperature under irradiation with 2x8W CFL lamps. Upon completion of reaction, determined by TLC, excess of solvent was evaporated under reduced pressure on the vacuum evaporator. Purification was performed using SiO_2 column chromatography with petrol ether/ethyl acetate as an eluents.

General procedure II, THIQ oxidation/Strecker reaction

Tetrahydroisoquinoline (0.25 mmol, 1 equiv.), $Ru(bpy)_3Cl_2*6H_2O$ (0.0025 mmol, 1 mol%), TMSCN (0.3 mmol, 1.2 equiv) were added to acetonitrile (1 mL) and stirred at room temperature under irradiation with 2x8W CFL lamps. Upon completion of reaction, determined by TLC, excess of solvent and reagents was evaporated under reduced pressure on the vacuum evaporator. Purification was performed using SiO_2 column chromatography with petrol ether/ethyl acetate as an eluents.

General procedure III, THIQ α-amino radical addition to Michael acceptors

Tetrahydroisoquinoline (0.25 mmol, 1 equiv.), $Ru(bpy)_3Cl_2*6H_2O$ (0.0025 mmol, 1 mol%), Trifluoroacetic acid (0.25 mmol, 1 equiv.) and α,β-unsaturated ketone (1 mmol, 4 equiv.) were added to CH_3CN (2 mL) in a flame dried round bottom flask. Solution was sparged with argon for 15 minutes to remove oxygen and stirred under irradiation of two 8W CFL lamps. Upon completion of the reaction, an excess of solvent was evaporated under reduced pressure on the vacuum evaporator. Purification was performed using SiO_2 thin layer chromatography with petrol ether/ethyl acetate as eluents.

1-(2-Phenyl-1,2,3,4-tetrahydroisoquinolin-1-yl)propan-2-one (2a)

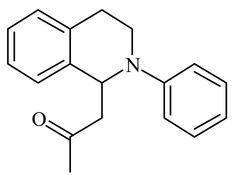

General procedure I was followed with 2-phenyl-1,2,3,4-tetrahydroisoquinoline (52.3 mg, 0.25 mmol), acetone (145.2 mg, 0.185 ml, 2.5 mmol), $Ru(bpy)_3Cl_2$ (1.9 mg, 0.0025 mmol), and L-proline (2.9

mg, 0.025 mmol) in CH_3CN. Crude reaction mixture was purified by column chromatography on silica gel (petrolether/ethylacetate = 7/1) to give the title compound (58 mg, 88 %) as yellowish oil.

Rf = 0.53 (Petrol Ether/EtOAc : 7/1); **^{1}H NMR** ($CDCl_3$, 500 MHz): δ 7.24 (t, 2H, ArH, J = 7.8 Hz), 7.12-7.15 (m, 4H, ArH), 6.93 (d, 2H, ArH, J = 8.5 Hz), 6.77 (t, 1H, J = 7.0 Hz), 5.39 (t, 1H, C(1)H, J = 6.2 Hz), 3.64 (dt, 1H, J = 5.5 Hz, 13.0 Hz), 3.52 (ddd, 1H, J = 4.4 Hz, 8.9 Hz, 12.5 Hz), 3.02-3.07 (m, 2H), 2.81 (dd, 2H, J = 7.2, 16.2 Hz), 2.06 (s, 3H, CH_3); **^{13}C NMR** ($CDCl_3$, 125.8 MHz): δ 207.2, 148.8, 138.3, 134.4, 129.3, 128.6, 126.83, 126.78, 126.3, 118.2, 114.8, 54.8, 50.2, 42.0, 31.1, 27.2.

Spectroscopic data are in agreement with the published data [S1].

1-(2-(4-Fluorophenyl)-1,2,3,4-tetrahydroisoquinolin-1-yl)propan-2-one (2b)

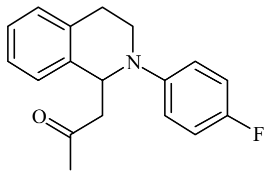

General procedure I was followed with 2-(4-fluorophenyl)-1,2,3,4-tetrahydroisoquinoline (69.3 mg, 0.25 mmol), acetone (145.2 mg, 0.185 ml, 2.5 mmol), $Ru(bpy)_3Cl_2$ (1.9 mg, 0.0025 mmol), and L-proline (2.9 mg, 0.025 mmol) in CH_3CN. Crude reaction mixture was purified by column chromatography on silica gel (petrolether/ethylacetate = 7/1) to give the title compound (67 mg, 95 %) as yellowish oil. **Rf** = 0.45 (Petrol Ether/EtOAc : 7/1); **^{1}H NMR** ($CDCl_3$, 500 MHz): δ 7.13-7.20 (m, 4H, ArH), 6.89-6.97 (m, 4H, ArH), 5.31 (t, 1H, C(1)H, J = 6.2 Hz), 3.59 (dt, 1H, J = 4.9, 12.5 Hz), 3.48-3.53 (m, 1H), 3.00-3.06 (m, 2H), 2.76-2.83 (m, 2H), 2.09 (s, 3H, CH_3); **^{13}C NMR** ($CDCl_3$, 125.8 MHz): δ 207.1, 156.4 (d, J = 237.6 Hz), 145.7, 138.0, 134.1, 128.8, 126.74, 126.72, 126.3, 117.1 (d, J = 7.6 Hz), 115.6 (d, J = 22.1 Hz), 55.5, 50.0, 42.6, 30.9, 26.7; Spectroscopic data are in agreement with the published data [S1].

1-(6,7-Dimethoxy-2-phenyl-1,2,3,4-tetrahydroisoquinolin-1-yl)propan-2-one (2c)

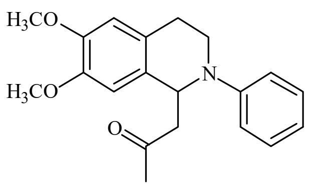

General procedure I was followed with 6,7-dimethoxy-2-phenyl-1,2,3,4-tetrahydroisoquinoline (67.3 mg, 0.25 mmol), acetone (145.2 mg, 0.185 ml, 2.5 mmol), $Ru(bpy)_3Cl_2$ (1.9 mg, 0.0025 mmol), and L-proline (2.9 mg, 0.025 mmol) in CH_3CN. Crude reaction mixture was purified by column chromatography on silica gel (petrolether/ethylacetate = 3/1) to give the title compound (77 mg, 95 %) as yellowish oil. **Rf** = 0.30 (Petrol Ether/EtOAc : 7/1); **^{1}H NMR** ($CDCl_3$, 500 MHz): δ 7.24 (t, 2H, ArH, J = 8.0 Hz), 6.93 (d, 2H, ArH, J = 8.0 Hz), 6.78

(t, 1H, ArH, *J* = 7.2 Hz), 6.69 (s, 1H, C(5)H or C(8)H), 6.61 (s, 1H, C(5)H or C(8)H), 5.30 (t, 1H, C(1)H, *J* = 6.2 Hz), 3.84 (s, 3H, OCH_3), 3.83 (s, 3H, OCH_3), 3.66 (dt, 1H, *J* = 5.0 Hz, 13.0 Hz), 3.49 (ddd, 1H, *J* = 4.5 Hz, 9.7 Hz, 12.8 Hz), 3.04 (dd, 1H, *J* = 5.5 Hz, 16.5 Hz), 2.97 (ddd, 1H, *J* = 5.8 Hz, 9.8 Hz, 15.8 Hz), 2.82 (dd, 1H, *J* = 6.8 Hz, 12.2 Hz), 2.70 (dt, 1H, *J* = 4.2 Hz, 16.0 Hz), 2.08 (s, 3H, $COCH_3$); **^{13}C NMR** ($CDCl_3$, 125.8 MHz): δ 207.6, 148.9, 147.7, 147.3, 130.1, 129.2, 126.2, 118.4, 115.1, 111.3, 109.7, 55.9, 55.8, 54.5, 50.1, 41.9, 31.1, 26.5;
Spectroscopic data are in agreement with the published data [S1].

1-(2-(4-fluorophenyl)-6,7-dimethoxy-1,2,3,4-tetrahydroisoquinolin-1-yl)propan-2-one (2d)

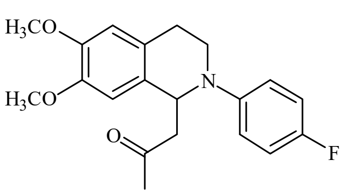

General procedure I was followed with 2-(4-fluorophenyl)-6,7-dimethoxy-1,2,3,4-tetrahydroisoquinoline (71.8 mg, 0.25 mmol), acetone (145.2 mg, 0.185 ml, 2.5 mmol), $Ru(bpy)_3Cl_2$ (1.9 mg, 0.0025 mmol), and L-proline (2.9 mg, 0.025 mmol) in CH_3CN. Crude reaction mixture was purified by column chromatography on silica gel (petrolether/ethylacetate = 3/1) to give the title compound (72 mg, 84 %) as yellowish oil. **Rf** = 0.36 (Petrol Ether/EtOAc : 3/1); **^{1}H NMR** ($CDCl_3$, 500 MHz): δ 6.86-6.94 (m, 4H, ArH), 6.66 (s, 1H, C(5)H or C(8)H), 6.59 (s, 1H, C(5)H or C(8)H), 5.18 (t, 1H, C(1)H, *J* = 6.2 Hz), 3.838 (s, 3H, OCH_3), 3.831 (s, 3H, OCH_3), 3.58 (ddd, 1H, *J* = 3.8 Hz, 5.2 Hz, 12.8 Hz), 3.46 (ddd, 1H, *J* = 4.2 Hz, 10.5 Hz, 16.0 Hz), 3.01 (dd, 1H, *J* = 6.2 Hz, 16.2 Hz), 2.92 (ddd, 1H, *J* = 5.8 Hz, 10.2 Hz, 16.0 Hz), 2.78 (dd, 1H, *J* = 6.5 Hz, 16.0 Hz), 2.64 (dt, 1H, *J* = 7.2 Hz, 16.0 Hz), 2.09 (s, 3H, $COCH_3$); **^{13}C NMR** ($CDCl_3$, 125.8 MHz): δ 207.5, 156.6 (d, *J* = 238.0 Hz), 147.8, 147.5, 145.9, 129.8, 126.0, 117.5 (d, *J* = 7.4 Hz), 115.6 (d, *J* = 22.4 Hz), 111.4, 109.6, 55.9, 55.8, 55.3, 50.0, 42.6, 31.0, 26.0;
Spectroscopic data are in agreement with the published data [S2].

1-(6,7-Dimethoxy-2-phenyl-1,2,3,4-tetrahydroisoquinolin-1-yl)butan-2-one (2e)

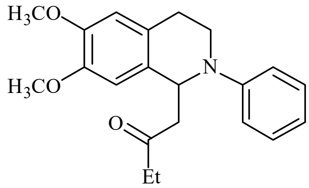

General procedure I was followed with 6,7-dimethoxy-2-phenyl-1,2,3,4-tetrahydroisoquinoline (67.3 mg, 0.25 mmol), ethyl methyl ketone (180.3 mg, 0.224 ml, 2.5 mmol), $Ru(bpy)_3Cl_2$ (1.9 mg, 0.0025 mmol), and L-proline (2.9 mg, 0.025 mmol) in CH_3CN. Crude reaction mixture was purified by column chromatography on silica gel (petrolether/ethylacetate = 3/1) to give the title compound (75.5 mg, 89 %) as yellowish oil. **Rf** = 0.29 (Petrol

Ether/EtOAc : 7/1); ^{1}**H NMR** ($CDCl_3$, 500 MHz): δ 7.24 (t, 2H, ArH, *J* = 8.2 Hz), 6.95 (d, 2H, ArH, *J* = 8.5 Hz), 6.78 (t, 1H, ArH, *J* = 7.0 Hz), 6.66 (s, 1H, C(5)H or C(8)H), 6.61 (s, 1H, C(5)H or C(8)H), 5.32 (t, 1H, C(1)H, *J* = 6.5 Hz), 3.85 (s, 3H, OCH_3), 3.83 (s, 3H, OCH_3), 3.66 (dt, 1H, *J* = 5.0 Hz, 13.0 Hz), 3.50 (ddd, 1H, *J* = 4.2 Hz, 9.2 Hz, 12.5 Hz), 3.02 (dd, 1H, *J* = 5.2 Hz, 15.8 Hz), 2.96-3.02 (m, 1H), 2.78 (dd, 1H, *J* = 7.0 Hz, 16.0 Hz), 2.72 (dt, 1H, *J* = 4.4 Hz, 16.0 Hz), 2.37 (dq, 1H, *J* = 7.4 Hz, 17.8 Hz), 2.28 (dq, 1H, *J* = 7.2 Hz, 17.8 Hz), 0.99 (t, 3H, $COCH_2C\underline{H}_3$, *J* = 7.2 Hz); ^{13}**C NMR** ($CDCl_3$, 125.8 MHz): δ 210.3, 148.9, 147.7, 147.3, 130.2, 129.3, 126.2, 118.2, 114.9, 111.3, 109.7, 55.9, 55.8, 54.9, 48.8, 41.8, 37.4, 26.6, 7.5; Spectroscopic data are in agreement with the published data [S3].

1-(6,7-Dimethoxy-2-(*p*-tolyl)-1,2,3,4-tetrahydroisoquinolin-1-yl)butan-2-one (2f)

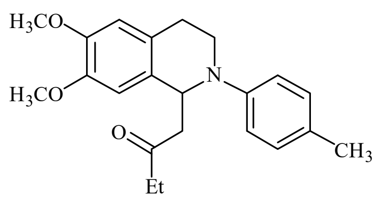

General procedure I was followed with 6,7-dimethoxy-2-(p-tolyl)-1,2,3,4-tetrahydroisoquinoline (70.8 mg, 0.25 mmol), ethyl methyl ketone (180.3 mg, 0.224 ml, 2.5 mmol), $Ru(bpy)_3Cl_2$ (1.9 mg, 0.0025 mmol), and L-proline (2.9 mg, 0.025 mmol) in CH_3CN. Crude reaction mixture was purified by column chromatography on silica gel (petrolether/ethylacetate = 3/1) to give the title compound (66 mg, 75 %) as yellowish oil. **Rf** = 0.52 (Petrol Ether/EtOAc : 3/1); ^{1}**H NMR** ($CDCl_3$, 500 MHz): δ 7.05 (d, 2H, ArH, *J* = 8.2 Hz), 6.86 (d, 2H, ArH, *J* = 8.2 Hz), 6.65 (s, 1H, C(5)H or C(8)H), 6.29 (s, 1H, C(5)H or C(8)H), 5.26 (t, 1H, C(1)H, *J* = 6.2 Hz), 3.84 (s, 3H, OCH_3), 3.83 (s, 3H, OCH_3), 3.63 (dt, 1H, *J* = 4.8 Hz, 12.0 Hz), 3.46 (ddd, 1H, *J* = 4.2 Hz, 10.2 Hz, 12.5 Hz), 2.94-3.01 (m, 2H), 2.75 (dd, 1H, *J* = 7.0 Hz, 15.5 Hz), 2.67 (brdt, 1H, *J* = 3.5 Hz, 16.0 Hz), 2.36 (dq, 1H, *J* = 7.3 Hz, 17.9 Hz), 2.28 (dq, partially hidden by $COCH_2C\underline{H}_3$ signal, 1H, *J* = 7.3 Hz, 17.9 Hz), 0.98 (t, 3H, $COCH_2C\underline{H}_3$, *J* = 7.5 Hz); ^{13}**C NMR** ($CDCl_3$, 125.8 MHz): δ 210.4, 147.7, 147.3, 147.0, 130.2, 129.8, 127.9, 126.2, 115.8, 111.3, 109.6, 55.9, 55.8, 55.3, 48.6, 41.9, 37.3, 26.5, 20.3, 7.5; Spectroscopic data are in agreement with the published data [S2].

1-Phenyl-2-(2-phenyl-1,2,3,4-tetrahydroisoquinolin-1-yl)ethanone (3a)

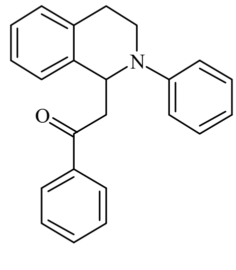

General procedure I was followed with 2-phenyl-1,2,3,4-tetrahydroisoquinoline (52.3 mg, 0.25 mmol), acetophenone (300.4 mg, 0.292 ml, 2.5 mmol), $Ru(bpy)_3Cl_2$ (1.9 mg, 0.0025 mmol), and L-proline (2.9 mg, 0.025 mmol) in CH_3CN. Crude reaction mixture was purified by column chromatography on silica gel (petrolether/ethylacetate = 7/1) to

give the title compound (68 mg, 83 %) as yellowish oil. **Rf** = 0.61 (Petrol Ether/EtOAc : 7/1); **^{1}H NMR** ($CDCl_3$, 500 MHz): δ 7.87 (d, 2H, ArH, *J* = 7.5 Hz), 7.54 (t, 1H, ArH, *J* = 7.2 Hz), 7.42 (t, 2H, ArH, *J* = 7.8 Hz), 7.11-7.27 (m, 7H, ArH), 6.99 (d, 1H, ArH, *J* = 8.0 Hz), 6.77 (t, 1H, ArH, *J* = 7.2 Hz), 5.69 (dd, 1H, C(1)H, *J* = 5.5 Hz, 7.0 Hz), 3.63-3.71 (m, 2H) 3.60 (dd, 1H, *J* = 4.8 Hz, 16.8 Hz), 3.42 (dd, 1H, *J* = 7.2, 16.8 Hz), 3.10-3.16 (m, 1H), 2.95 (dt, 1H, *J* = 5.0 Hz, 16.0 Hz); **^{13}C NMR** ($CDCl_3$, 125.8 MHz): δ 198.6, 148.7, 138.5, 137.2, 134.5, 133.1, 129.3, 128.5, 128.1, 127.1, 126.8, 126.2, 117.9, 114.3, 55.0, 45.3, 42.1, 27.5; Spectroscopic data are in agreement with the published data [S4].

2-(2-(4-methoxyphenyl)-1,2,3,4-tetrahydroisoquinolin-1-yl)-1-phenylethanone (3b)

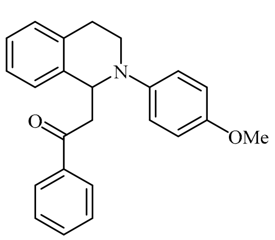

General procedure I was followed with 2-(4-methoxyphenyl)-1,2,3,4-tetrahydroisoquinoline (60 mg, 0.25 mmol), acetophenone (300.4 mg, 0.292 ml, 2.5 mmol), Ru(bpy)$_3$Cl$_2$ (1.9 mg, 0.0025 mmol), and L-proline (2.9 mg, 0.025 mmol) in MeOH/CH_3CN=1/1 (2 mL). Crude reaction mixture was purified by column chromatography on silica gel (petrolether/ethylacetate = 7/1) to give the title compound (62.5 mg, 70 %) as yellowish solid. **Rf** = 0.66 (Petrol Ether/EtOAc : 7/1); **^{1}H NMR** ($CDCl_3$, 400 MHz): δ 7.82 (d, *J* = 7.3 Hz, 2H), 7.50 (t, *J* = 7.4 Hz, 1H), 7.38 (t, *J* = 7.7 Hz, 2H), 7.21 – 7.05 (m, 4H), 6.92 (d, *J* = 9.1 Hz, 2H), 6.78 (d, *J* = 9.1 Hz, 2H), 5.51 (t, *J* = 6.0 Hz, 1H), 3.71 (s, 3H), 3.60-3.50 (m, 3H), 3.28 (dd, *J* = 16.3, 6.6 Hz, 1H), 3.13 – 3.01 (m, 1H), 2.82 (dt, *J* = 16.3, 4.1 Hz, 1H)č **^{13}C NMR** ($CDCl_3$, 101 MHz): δ 198.7, 152.9, 143.6, 138.5, 137.3, 134.3, 132.9, 128.8, 128.4, 128.1, 127.0, 126.6, 126.1, 117.8, 114.6, 56.2, 55.6, 44.9, 42.7, 27.2.

Spectroscopic data are in agreement with the published data [S5].

2-(6,7-dimethoxy-2-phenyl-1,2,3,4-tetrahydroisoquinolin-1-yl)-1-phenylethanone (3c)

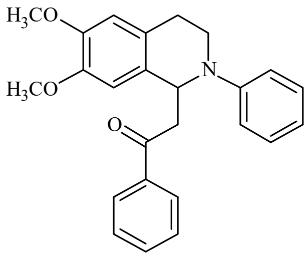

General procedure I was followed with 6,7-dimethoxy-2-phenyl-1,2,3,4-tetrahydroisoquinoline (67.3 mg, 0.25 mmol), acetophenone (300.4 mg, 0.292 ml, 2.5 mmol), Ru(bpy)$_3$Cl$_2$ (1.9 mg, 0.0025 mmol), and L-proline (2.9 mg, 0.025 mmol) in CH_3CN. Crude reaction mixture was purified by column chromatography on silica gel (petrolether/ethylacetate = 3/1) to give the title compound (71 mg, 73 %) as yellowish oil. **Rf** = 0.29 (Petrol Ether/EtOAc : 3/1); **^{1}H NMR** ($CDCl_3$, 500 MHz): δ 7.85 (d, 2H, ArH, *J* = 7.5 Hz), 7.52 (t, 1H, ArH, *J* = 7.5 Hz), 7.41 (t, 2H, ArH, *J* = 7.8 Hz),

7.24 (t, 2H, ArH, $J = 7.5$ Hz), 6.98 (d, 2H, ArH, $J = 8.5$ Hz), 6.76 (t, 1H, ArH, $J = 7.2$ Hz), 6.70 (s, 1H, C(5)H or C(8)H), 6.63 (s, 1H, C(5)H or C(8)H), 5.36 (dd, 1H, C(1)H, $J = 5.0$ Hz, 7.0 Hz), 3.84 (s, 3H, OCH_3), 3.74 (s, 3H, OCH_3), 3.68 (dt, 1H, $J = 5.2$ Hz, 12.5 Hz), 3.32-3.61 (m, 2H), 3.39 (dd, 1H, $J = 7.5$ Hz, 16.0 Hz), 3.03 (ddd, 1H, $J = 5.8$ Hz, 8.4 Hz, 15.6 Hz), 2.81 (dt, 1H, $J = 4.4$ Hz, 16.0 Hz); **^{13}C NMR** ($CDCl_3$, 125.8 MHz): δ 199.1, 148.9, 147.8, 147.3, 137.3, 133.1, 130.4, 129.3, 128.5, 128.1, 126.3, 118.1, 114.7, 111.3, 110.1, 55.9 (2C overlapped), 55.0, 45.1, 42.0, 27.0; Spectroscopic data are in agreement with the published data [S6].

2-(2-(4-Fluorophenyl)-6,7-dimethoxy-1,2,3,4-tetrahydroisoquinolin-1-yl)-1-phenylethanone (3d)

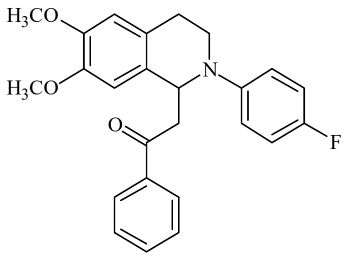

General procedure I was followed with 2-(4-fluorophenyl)-6,7-dimethoxy-1,2,3,4-tetrahydroisoquinoline (71.8 mg, 0.25 mmol), acetophenone (300.4 mg, 0.292 ml, 2.5 mmol), $Ru(bpy)_3Cl_2$ (1.9 mg, 0.0025 mmol), and L-proline (2.9 mg, 0.025 mmol) in CH_3CN. Crude reaction mixture was purified by column chromatography on silica gel (petrolether/ethylacetate = 3/1) to give the title compound (74 mg, 73 %) as yellowish oil. **Rf** = 0.34 (Petrol Ether/EtOAc : 7/1); **^{1}H NMR** ($CDCl_3$, 500 MHz): δ 7.85 (d, 2H, ArH, $J = 7.5$ Hz), 7.53 (d, 1H, ArH, $J = 7.5$ Hz), 7.41 (t, 2H, ArH, $J = 7.5$ Hz), 6.92 (d, 4H, ArH, $J = 6.5$ Hz), 6.67 (s, 1H, C(5)H or C(8)H), 6.62 (s, 1H, C(5)H or C(8)H), 5.44 (t, 1H, C(1)H, $J = 6.0$ Hz), 3.84 (s, 3H, OCH_3), 3.74 (s, 3H, OCH_3), 3.52-3.63 (m, 3H), 3.33 (dd, 1H, $J = 6.8$ Hz, 16.2 Hz), 3.00 (ddd, 1H, $J = 6.1$ Hz, 9.6 Hz, 15.9 Hz), 2.74 (brd, 1H, $J = 16.0$ Hz); **^{13}C NMR** ($CDCl_3$, 125.8 MHz): δ 199.0, 156.4 (d, $J = 237.5$ Hz), 147.8, 147.4, 145.8, 137.3, 133.2, 130.1, 128.6, 128.1, 126.1, 117.1 (d, $J = 7.3$ Hz), 115.6 (d, $J = 22.1$ Hz), 111.4, 109.9, 55.9 (3C overlapped), 44.9, 42.6.3, 26.6; Spectroscopic data are in agreement with the published data [S2].

1-(6,7-Dimethoxy-2-(*p*-tolyl)-1,2,3,4-tetrahydroisoquinolin-1-yl)butan-2-one (3e)

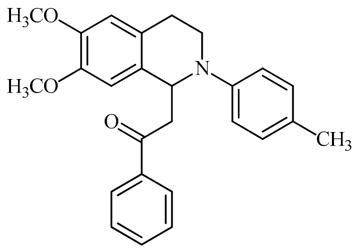

General procedure I was followed with 6,7-dimethoxy-2-(p-tolyl)-1,2,3,4-tetrahydroisoquinoline (70.8 mg, 0.25 mmol), ethyl methyl ketone (180.3 mg, 0.224 ml, 2.5 mmol), $Ru(bpy)_3Cl_2$ (1.9 mg, 0.0025 mmol), and L-proline (2.9 mg, 0.025 mmol) in MeOH (2 mL). Crude reaction mixture was

purified by column chromatography on silica gel (petrolether/ethylacetate = 3/1) to give the title compound (71 mg, 71 %) as yellowish oil. **Rf** = 0.53 (Petrol Ether/EtOAc : 3/1); **^{1}H NMR** ($CDCl_3$, 400 MHz): δ 7.84 (d, *J* = 8.2 Hz, 2H), 7.52 (t, *J* = 7.4 Hz, 1H), 7.40 (t, *J* = 7.7 Hz, 2H), 7.04 (d, *J* = 8.4 Hz, 2H), 6.90 (d, *J* = 8.4 Hz, 2H), 6.69 (s, 1H), 6.61 (s, 1H), 5.50 (t, *J* = 4 Hz, 1H), 3.83 (s, 3H), 3.73 (s, 3H), 3.69 – 3.60 (m, 1H), 3.58 – 3.46 (m, 2H), 3.35 (dd, *J* = 16.1, 7.2 Hz, 1H), 3.02 (ddd, *J* = 15.6, 9.6, 5.7 Hz, 1H), 2.76 (dt, *J* = 16.0, 4.3 Hz, 1H), 2.23 (s, 3H); **^{13}C NMR** ($CDCl_3$, 101 MHz): δ 199.2, 147.7, 147.2, 146.9, 137.4, 133.0, 130.4, 129.8, 128.5, 128.1, 127.8, 126.2, 115.5, 111.3, 109.9, 55.8, 55.4, 44.8, 42.1, 26.9, 20.3; **IR** (ATR): ν = 2987 (m), 2834 (m), 1679 (s), 1612 (m), 1514 (vs), 1449 (m), 1273 (s), 1249 (s), 1116 (m), 1021 (m); **HRMS**: m/z (ESI/TOF) calc for $C_{26}H_{27}NO_3$ (M+) 401.1991, found 401.1990;

1-Phenyl-3-(2-phenyl-1,2,3,4-tetrahydroisoquinolin-1-yl)propan-1-one (3f)

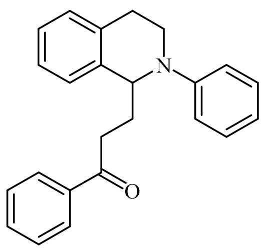

General procedure III was followed with 2-phenyl-1,2,3,4-tetrahydroisoquinoline (52.3 mg, 0.25 mmol), phenyl vinyl ketone (132 mg, 0.132 ml, 1 mmol), $Ru(bpy)_3Cl_2$ (1.9 mg, 0.0025 mmol), and TFA (19 µL, 0.025 mmol) in CH_3CN (2 mL). Crude reaction mixture was purified by preparative thin layer chromatography on silica gel (petrolether/ethylacetate = 7/1) to give the title compound (66 mg, 77 %) as yellowish oil. **^{1}H NMR ($CDCl_3$, 400 MHz)**: δ 7.89 (d, 2H, ArH, *J* = 8.4 Hz), 7.51 (t, 1H, ArH, *J* = 7.4 Hz), 7.40 (t, 2H, ArH, *J* = 7.4 Hz), 7.17 (dd, 5H, ArH, *J* = 6.8 Hz, 18.6 Hz), 7.08-7.13 (m, 1H, ArH), 6.88 (d, 2H, ArH, *J* = 8.4 Hz), 6.70 (t, 1H, ArH, *J* = 7.2 Hz), 4.83 (dd, 1H, C(1)H, *J* = 6.0 Hz, 8.8 Hz), 3.61 (dd, 2H, *J* = 4.4 Hz, 8.8 Hz), 3.10 (td, 2H, *J* = 2.0 Hz, 6.8 Hz), 2.96-3.04 (m, 1H), 2.76 (dt, 1H, *J* = 4.5 Hz, 16.0 Hz), 2.40 (dt, 1H, *J* = 7.1 Hz, 14.9 Hz), 2.22 (dt, 1H, *J* = 6.9 Hz, 13.8 Hz); **^{13}C NMR ($CDCl_3$, 100 MHz)**: δ 200.1, 149.8, 138.4, 137.0, 134.9, 132.9, 129.2, 128.7, 128.5, 128.0, 127.3, 126.5, 125.9, 117.7, 114.7, 58.2, 41.6, 35.4, 30.9, 26.4; **IR (ATR)**: ν = 3059 (m), 3023 (m), 2930 (m), 1682 (vs), 1598 (vs), 1504 (vs), 1449 (m), 1394 (m), 1326 (w), 1271 (w), 1233 (m), 1000 (w) 751 (s), 692 (m) cm^{-1}; Spectroscopic data are in agreement with the published data [S7].

3-(6,7-Dimethoxy-2-phenyl-1,2,3,4-tetrahydroisoquinolin-1-yl)-1-phenylpropan-1-one (3g)

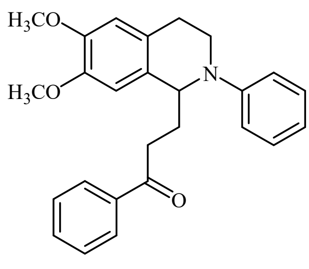

General procedure III was followed with 6,7-dimethoxy-2-phenyl-1,2,3,4-tetrahydroisoquinoline (67.3 mg, 0.25 mmol), phenyl vinyl ketone (132 mg, 0.132 ml, 1 mmol), Ru(bpy)$_3$Cl$_2$ (1.9 mg, 0.0025 mmol), and TFA (19 μL, 0.025 mmol) in CH_3CN (2 mL). Crude reaction mixture was purified by preparative thin layer chromatography on silica gel (petrolether/ethylacetate = 7/1) to give the title compound (83 mg, 83 %) as yellowish oil.

^{1}H NMR (CDCl$_3$, 400 MHz): δ 7.91 (d, 2H, ArH, *J* = 7.6 Hz), 7.53 (t, 1H, ArH, *J* = 7.4 Hz), 7.42 (t, 2H, ArH, *J* = 7.6 Hz), 7.17 (t, 2H, ArH, *J* = 8.0 Hz), 6.88 (d, 2H, ArH, *J* = 8.4 Hz), 6.71 (t, 1H, ArH, *J* = 7.2 Hz), 6.69 (s, 1H, ArH), 6.58 (s, 1H, ArH), 4.75 (dd, 1H, C(1)H, *J* = 5.2 Hz, 9.2 Hz), 3.84 (s, 3H, OCH_3), 3.82 (s, 3H, OCH_3), 3.65 (dt, 1H, *J* = 4.8 Hz, 13.2 Hz), 3.57 (ddd, 1H, *J* = 4.1 Hz, 9.7 Hz, 13.7 Hz), 3.12 (td, 2H, *J* = 2.7 Hz, 6.7 Hz), 2.93 (ddd, 1H, *J* = 5.7 Hz, 9.9 Hz, 15.7 Hz), 2.63 (dt, 1H, *J* = 4.1 Hz, 16.0 Hz), 2.37 (dt, 1H, *J* = 6.0 Hz, 15.2 Hz), 2.25 (dt, 1H, *J* = 7.6 Hz, 13.5 Hz); **^{13}C NMR (CDCl$_3$, 100 MHz)**: δ 200.3, 150.0, 147.6, 147.2, 137.0, 132.9, 130.3, 129.2, 128.5, 128.0, 126.7, 117.9, 115.0, 114.5, 110.2, 57.8, 55.94, 55.86, 41.6, 33.4, 30.8, 25.7; **IR (ATR)**: ν = 3059 (w), 2934 (m), 2834 (w), 1682 (vs), 1598 (vs), 1516 (vs), 1504 (vs), 1449 (m), 1390 (m), 1357 (m), 1249 (vs), 1113 (s) 1031 (m), 991 (m), 911 (w), 859 (w), 750 (s), 693 (m) cm^{-1}; **HRMS**: m/z (HESI) calc for $C_{26}H_{28}NO_3$ $[M+H]^+$ 402.2064, found 402.2053.

3-(2-(4-Fluorophenyl)-6,7-dimethoxy-1,2,3,4-tetrahydroisoquinolin-1-yl)-1-phenylpropan-1-one (3h)

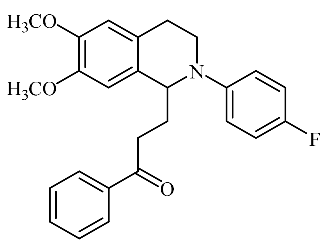

General procedure was followed with 2-(4-fluorophenyl)-6,7-dimethoxy-1,2,3,4-tetrahydroisoquinoline (71.8 mg, 0.25 mmol), phenyl vinyl ketone (132 mg, 0.132 ml, 1 mmol), Ru(bpy)$_3$Cl$_2$ (1.9 mg, 0.0025 mmol), and TFA (19 μL, 0.025 mmol) in CH_3CN (2 mL). Crude reaction mixture was purified by preparative thin layer chromatography on silica gel (petrolether/ethylacetate = 7/1) to give the title compound (77 mg, 73 %) as yellowish oil.

^{1}H NMR (CDCl$_3$, 500 MHz): δ 7.90 (d, 2H, ArH, *J* = 8.2 Hz), 7.53 (t, 1H, ArH, *J* = 7.2 Hz), 7.42 (t, 2H, ArH, *J* = 7.8 Hz), 6.85 (t, 2H, ArH, *J* = 8.8 Hz), 6.79 (dd, 2H, ArH, *J* = 4.5 Hz, 9.0 Hz), 6.69 (s, 1H, ArH), 6.57 (s, 1H, ArH), 4.58 (dd, 1H, C(1)H, *J* = 5.0 Hz, 9.0 Hz), 3.84 (s, 3H, OCH_3), 3.83 (s, 3H, OCH_3), 3.47-3.57 (m, 2H), 3.04-3.16 (m, 2H), 2.84 (ddd, 1H, *J* = 6.2

Hz, 9.8 Hz, 16.0 Hz), 2.58 (dt, 1H, *J* = 4.1 Hz, 16.0 Hz), 2.35 (ddt, 1H, *J* = 6.5 Hz, 9.3 Hz, 13.4 Hz), 2.19-2.26 (m, 1H); **^{13}C NMR ($CDCl_3$, 125.8 MHz)**: δ 200.3, 156.3 (d, *J* = 237.6 Hz), 147.7, 147.4, 146.85, 137.1, 132.9, 130.0, 128.5, 128.0, 126.6, 117.6 (d, *J* = 7.3 Hz), 115.5 (d, *J* = 22.0 Hz), 111.5, 110.1, 58.4, 55.9, 55.8, 42.8, 35.4, 31.0, 25.3; **IR (ATR)**: ν = 3067 (w), 2997 (w), 2934 (m), 2835 (w), 1681 (s), 1610 (w), 1510 (vs), 1449 (m), 1390 (w), 1355 (w), 1248 (s), 1231 (s), 1113 (m) 1028 (w), 1001 (w), 817 (w), 748 (w), 691 (w) cm^{-1}; **HRMS**: m/z (HESI) calc for $C_{26}H_{27}FNO_3$ $[M+H]^+$ 420.1970, found 420.1960.

1-Phenyl-5-(2-phenyl-1,2,3,4-tetrahydroisoquinolin-1-yl)pentan-3-one (**3i**)

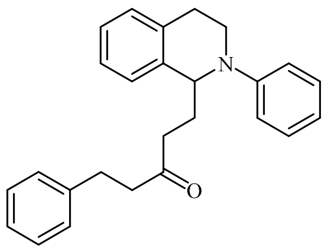

General procedure III was followed with 2-phenyl-1,2,3,4-tetrahydroisoquinoline (52.3 mg, 0.25 mmol), 5-phenylpent-1-en-3-one (160 mg, 1 mmol), $Ru(bpy)_3Cl_2$ (1.9 mg, 0.0025 mmol), and TFA (19 μL, 0.025 mmol) in CH_3CN (2 mL). Crude reaction mixture was purified by preparative thin layer chromatography on silica gel (petrolether/ethylacetate = 7/1) to give the title compound (77 mg, 83 %) as yellowish oil. **^{1}H NMR ($CDCl_3$, 400 MHz)**: δ 7.16-7.26 (m, 5H, ArH), 7.06-7.15 (m, 6H, ArH), 6.86 (d, 2H, ArH, *J* = 8.4 Hz), 6.73 (t, 1H, ArH, *J* = 7.2 Hz), 4.71 (dd, 1H, C(1)H, *J* = 5.6 Hz, 9.2 Hz), 3.58 (dt, 1H, *J* = 5.2 Hz, 13.2 Hz), 3.51 (ddd, 1H, *J* = 4.3 Hz, 9.5 Hz, 13.5 Hz), 2.97 (ddd, 1H, *J* = 5.9 Hz, 9.7 Hz, 15.9 Hz), 2.84 (t, 2H, *J* = 7.6 Hz), 2.72 (dt, 1H, *J* = 4.4 Hz, 16.4 Hz), 2.65 (dt, 1H, *J* = 4.8 Hz, 7.6 Hz), 2.50 (t, 2H, *J* = 6.8 Hz), 2.24 (ddd, 1H, *J* = 6.6 Hz, 8.9 Hz, 21.3 Hz), 2.04 (dt, 1H, *J* = 7.6 Hz, 13.6 Hz); **^{13}C NMR ($CDCl_3$, 100 MHz)**: δ 209.6, 149.8, 141.1, 138.3, 134.8, 129.3, 128.7, 128.4, 128.3, 127.2, 126.5, 126.0, 125.9, 117.7, 114.6, 57.9, 44.6, 41.4, 39.6, 30.4, 29.7, 26.3; **IR (ATR)**: ν = 3060 (m), 3025 (m), 2927 (m), 1712 (vs), 1598 (vs), 1504 (vs), 1453 (m), 1395 (m), 1326 (w), 1219 (m), 1156 (w), 1032 (w) 750 (s), 698 (m) cm^{-1}; Spectroscopic data are in agreement with the published data [S8].

1-(2-(4-Fluorophenyl)-1,2,3,4-tetrahydroisoquinolin-1-yl)-5-phenylpentan-3-one (**3j**)

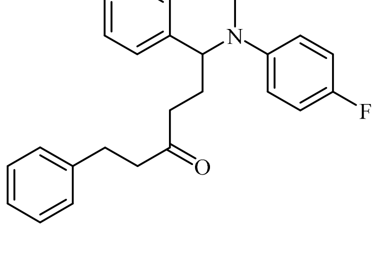

General procedure III was followed with 2-(4-fluorophenyl)-1,2,3,4-tetrahydroisoquinoline (56.8 mg, 0.25 mmol), 5-phenylpent-1-en-3-one (160 mg, 1 mmol), $Ru(bpy)_3Cl_2$ (1.9 mg, 0.0025 mmol), and TFA (19 μL, 0.025 mmol) in CH_3CN (2 mL). Crude reaction mixture was purified by preparative thin layer

chromatography on silica gel (petrolether/ethylacetate = 7/1) to give the title compound (64 mg, 66 %) as yellowish amorphous solid.

^{1}H NMR ($CDCl_3$, 400 MHz): δ 7.23-7.27 (m, 2H, ArH), 7.06-7.19 (m, 7H, ArH), 6.89 (d, 2H, ArH, *J* = 8.8 Hz), 6.78 (dd, 1H, ArH, *J* = 4.6 Hz, 9.0 Hz), 4.55 (dd, 1H, C(1)H, *J* = 5.6 Hz, 9.2 Hz), 3.49 (dd, 1H, *J* = 4.6 Hz, 9.4 Hz), 2.89-2.49 (m, 1H), 2.85 (t, 2H, *J* = 7.6 Hz), 2.64-2.71 (m, 3H), 2.49 (t, 2H, *J* = 6.6 Hz), 2.21 (dt, 1H, *J* = 6.4 Hz, 15.2 Hz), 2.01 (dt, 1H, *J* = 7.6 Hz, 13.4 Hz); **^{13}C NMR ($CDCl_3$, 100 MHz)**: δ 209.6, 156.2 (d, *J* = 236.2 Hz), 146.6 (d, *J* = 2.1 Hz), 141.1, 138.1, 134.7, 128.8, 128.4, 128.3, 127.2, 126.5, 126.04, 125.99, 116.9 (d, *J* = 7.3 Hz), 115.6 (d, *J* = 21.9 Hz), 58.5, 44.6, 42.5, 39.6, 30.5, 29.7, 25.9; **IR (ATR)**: ν = 3060 (w), 3026 (w), 2925 (m), 1710 (s), 1604 (w), 1508 (vs), 1453 (m), 1395 (w), 1332 (w), 1231 (s), 1162 (w), 818 (m) 750 (m), 700 (m) cm^{-1}; **HRMS**: m/z (HESI) calc for $C_{26}H_{27}FNO$ $[M+H]^+$ 388.2071, found 388.2063.

1-(6,7-Dimethoxy-2-phenyl-1,2,3,4-tetrahydroisoquinolin-1-yl)-5-phenylpentan-3-one (3k)

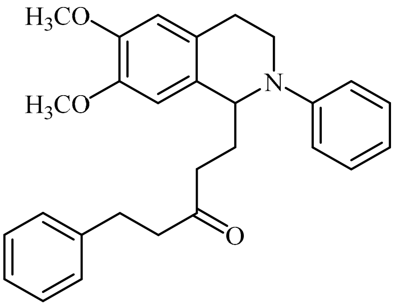

General procedure III was followed with 6,7-dimethoxy-2-phenyl-1,2,3,4-tetrahydroisoquinoline (67.3 mg, 0.25 mmol), 5-phenylpent-1-en-3-one (160 mg, 1 mmol), $Ru(bpy)_3Cl_2$ (1.9 mg, 0.0025 mmol), and TFA (19 µL, 0.025 mmol) in CH_3CN (2 mL). Crude reaction mixture was purified by preparative thin layer chromatography on silica gel (petrolether/ethylacetate = 7/1) to give the title compound (82 mg, 76 %) as yellowish oil.

^{1}H NMR ($CDCl_3$, 500 MHz): δ 7.15-7.26 (m, 5H, ArH), 7.10 (d, 2H, ArH, *J* = 7.0 Hz), 6.85 (d, 2H, ArH, *J* = 8.0 Hz), 6.74 (t, 1H, ArH, *J* = 7.2 Hz), 6.65 (s, 1H, ArH), 6.55 (s, 1H, ArH), 4.63 (dd, 1H, C(1)H, *J* = 4.8 Hz, 9.8 Hz), 3.86 (s, 3H, OCH_3), 3.82 (s, 3H, OCH_3), 3.62 (ddd, 1H, *J* = 4.0 Hz, 5.0 Hz, 13.5 Hz) 3.44-5.50 (m, 1H), 2.90 (ddd, 1H, *J* = 5.6 Hz, 10.6 Hz, 16.1 Hz), 2.84 (t, 2H, *J* = 8.0 Hz), 2.63-2.67 (m, 2H), 2.51-2.59 (m, 3H), 2.15-2.22 (m, 1H), 2.04-2.12 (m, 1H); **^{13}C NMR ($CDCl_3$, 100 MHz)**: δ 209.8, 150.0, 147.6, 147.2, 141.1, 130.3, 129.3,

128.4, 128.3, 126.6, 126.0, 118.0, 115.2, 111.5, 110.1, 57.6, 56.0, 55.8, 44.6, 41.2, 39.6, 30.4, 29.7, 25.5; **IR (ATR)**: ν = 3026 (w), 2999 (w), 2993 (m), 2834 (w), 1710 (s), 1597 (s), 1516 (vs), 1503 (vs), 1464 (m), 1408 (m), 1356 (m), 1250 (s), 1112 (s) 1031 (m), 989 (w), 860 (w), 751 (m), 699 (m) cm^{-1}; **HRMS**: m/z (HESI) calc for $C_{28}H_{32}NO_3$ $[M+H]^+$ 430.2377, found 430.2368.

1-(2-(4-Fluorophenyl)-6,7-dimethoxy-1,2,3,4-tetrahydroisoquinolin-1-yl)-5 phenylpentan-3-one (**3l**)

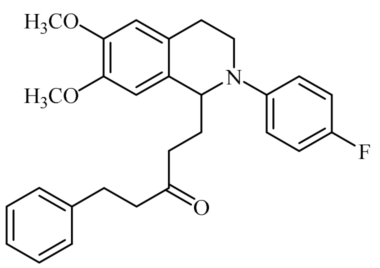

General procedure III was followed with 2-(4-fluorophenyl)-6,7-dimethoxy-1,2,3,4-tetrahydroisoquinoline (71.8 mg, 0.25 mmol), 5-phenylpent-1-en-3-one (160 mg, 1 mmol), $Ru(bpy)_3Cl_2$ (1.9 mg, 0.0025 mmol), and TFA (19 μL, 0.025 mmol) in CH_3CN (2 mL). Crude reaction mixture was purified by preparative thin layer chromatography on silica gel (petrolether/ethylacetate = 7/1) to give the title compound (77 mg, 69 %) as yellowish amorphous solid.

^{1}H NMR ($CDCl_3$, 400 MHz): δ 7.25 (t, 2H, ArH, *J* = 7.4 Hz), 7.17 (t, 1H, ArH, *J* = 7.4 Hz), 7.11 (d, 2H, ArH, *J* = 7.2 Hz), 6.88 (t, 2H, ArH, *J* = 8.6 Hz), 6.78 (dd, 1H, ArH, *J* = 4.4 Hz, 9.2 Hz), 6.65 (s, 1H, ArH), 6.54 (s, 1H, ArH), 4.45 (dd, 1H, C(1)H, *J* = 4.8 Hz, 9.6 Hz), 3.87 (s, 3H, OCH_3), 3.83 (s, 3H, OCH_3), 3.45-3.50 (m, 2H), 2.85 (t, 2H, *J* = 7.6 Hz), 2.77-2.81 (m, 1H), 2.67 (dt, 2H, *J* = 3.6 Hz, 7.5 Hz), 2.50-2.54 (m, 3H), 2.13-2.20 (m, 1H), 2.01-2.10 (m, 1H); **^{13}C NMR ($CDCl_3$, 100 MHz)**: δ 209.8, 156.3 (d, *J* = 236.5 Hz), 147.6, 147.3, 146.8 (d, *J* = 2.1 Hz), 141.0, 130.0, 128.4, 128.2, 126.5, 126.0, 117.6 (d, *J* = 7.4 Hz), 115.6 (d, *J* = 21.9 Hz), 111.5, 110.0, 58.2, 56.0, 55.8, 44.7, 42.5, 39.6, 30.5, 29.7, 25.0. **IR (ATR)**: ν = 3026 (w), 2934 (m), 2834 (w), 1710 (s), 1609 (w), 1508 (vs), 1453 (m), 1355 (w), 1250 (s), 1230 (s), 1112 (s), 1030 (w), 819 (m), 751 (w), 701 (m) cm^{-1}; **HRMS**: m/z (HESI) calc for $C_{28}H_{31}FNO_3$ $[M+H]^+$ 448.2285, found 448.2273.

2-Phenyl-1,2,3,4-tetrahydroisoquinoline-1-carbonitrile (4a)

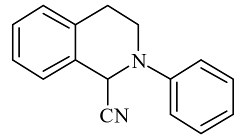

General procedure II was followed with 2-phenyl-1,2,3,4-tetrahydroisoquinoline (52.3 mg, 0.25 mmol), trimethylsilyl cyanide (29.8 mg, 0.038 ml, 0.3 mmol) and $Ru(bpy)_3Cl_2$ (1.9 mg, 0.0025 mmol), in CH_3CN (1 mL). Crude reaction mixture was purified by column chromatography on silica gel (petrolether/ethylacetate = 7/1) to give the title compound (56 mg, 95 %) as colourless solid.

Rf = 0.44 (Petrol Ether/EtOAc : 7/1); mp: 99-101 °C; **^{1}H NMR** ($CDCl_3$, 500 MHz): δ 7.36 (t, 2H, ArH, *J* = 8.0 Hz), 7.22-7.32 (m, 4H, ArH), 7.08 (d, 2H, ArH, *J* = 8.0 Hz), 7.02 (t, 1H, ArH, *J* = 7.2 Hz), 5.51 (s, 1H, C(1)H), 3.76 (dddd, 1H, *J* = 1.0 Hz, 3.0 Hz, 6.0 Hz, 12.5 Hz), 3.47 (ddd, 1H, *J* = 3.9 Hz, 10.9 Hz, 12.1 Hz), 3.14 (ddd, 1H, *J* = 5.9 Hz, 10.6 Hz, 16.4 Hz), 2.95 (dt, 1H, *J* = 3.4 Hz, 16.5 Hz); **^{13}C NMR** ($CDCl_3$, 125.8 MHz): δ 148.3, 134.5, 129.5 (2C overlapped), 129.3, 128.7, 127.0, 126.8, 121.8, 117.7, 117.5, 53.1, 44.1, 28.4; Spectroscopic data are in agreement with the published data [S9].

2-(4-Fluorophenyl)-6,7-dimethoxy-1,2,3,4-tetrahydroisoquinoline-1-carbonitrile (4b)

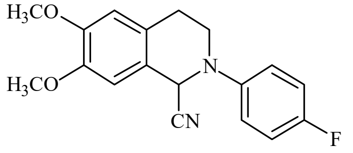

General procedure II was followed with 2-(4-fluorophenyl)-6,7-dimethoxy-1,2,3,4-tetrahydroisoquinoline (71.8 mg, 0.25 mmol), trimethylsilyl cyanide (29.8 mg, 0.038 ml, 0.3 mmol), and $Ru(bpy)_3Cl_2$ (1.9 mg, 0.0025 mmol), in CH_3CN (1 mL). Crude reaction mixture was purified by column chromatography on silica gel (petrolether/ethylacetate = 3/1) to give the title compound (71 mg, 91 %) as colourless solid. **Rf** = 0.44 (Petrol Ether/EtOAc : 3/1); mp: 114-116 °C; **^{1}H NMR** ($CDCl_3$, 500 MHz): δ 7.05-7.06 (m, 4H, ArH), 6.72 (s, 1H, C(5)H or C(8)H), 6.68 (s, 1H, C(5)H or C(8)H), 5.33 (s, 1H, C(1)H), 3.882 (s, 3H, OCH_3), 3.876 (s, 3H, OCH_3), 3.61 (brdd, 1H, *J* = 5.5 Hz, 12.5 Hz), 3.41 (dt, 1H, *J* = 4.0 Hz, 11.8 Hz), 3.08 (ddd, 1H, *J* = 5.8 Hz, 10.8 Hz, 16.2 Hz), 2.83 (dt, 1H, *J* = 2.5 Hz, 15.0 Hz); **^{13}C NMR** ($CDCl_3$, 125.8 MHz): δ 158.6 (d, *J* = 242.2 Hz), 149.4, 148.1, 145.1 (d, *J* = 2.1 Hz), 126.5, 120.9, 120.5 (d, *J* = 8.1 Hz), 117.6, 116.1 (d, *J* = 22.4 Hz), 111.5, 109.3, 56.0, 55.9, 54.5, 44.8, 28.1. Spectroscopic data are in agreement with the published data [S2].

6,7-Dimethoxy-2-(*p*-tolyl)-1,2,3,4-tetrahydroisoquinoline-1-carbonitrile (4c)

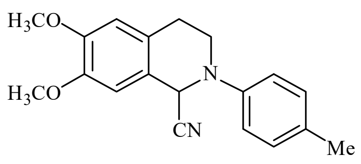

General procedure II was followed with 6,7-dimethoxy-2-(p-tolyl)-1,2,3,4-tetrahydroisoquinoline (70.8 mg, 0.25 mmol), trimethylsilyl cyanide (29.8 mg, 0.038 ml, 0.3 mmol), and $Ru(bpy)_3Cl_2$ (1.9 mg, 0.0025 mmol), in CH_3CN (1 mL). Crude reaction mixture was purified by column chromatography on silica gel (petrolether/ethylacetate = 3/1) to give the title compound (65.5 mg, 85 %) as colourless amorphous solid. **Rf** = 0.53 (Petrol Ether/EtOAc : 3/1); **^{1}H NMR** ($CDCl_3$, 500 MHz): δ 7.16 (d, 2H, ArH, *J* = 8.5 Hz), 6.99 (d, 2H, ArH, *J* = 8.5 Hz), 6.73 (s, 1H, C(5)H or C(8)H), 6.67 (s, 1H, C(5)H or C(8)H), 5.38 (s, 1H, C(1)H), 3.88 (s, 6H, $2OCH_3$), 3.69 (brdd, 1H, *J* = 5.5 Hz, 12.5 Hz), 3.40 (td, 1H, *J* = 3.8 Hz, 10.9 Hz), 3.07 (ddd, 1H, *J* = 5.8 Hz, 11.0 Hz, 16.2 Hz), 2.82 (brdt, 1H, *J* = 2.2 Hz, 15.5

Hz), 2.31 (s, 3H, ArC$\underline{H}_3$); **^{13}C NMR** ($CDCl_3$, 125.8 MHz): δ 149.3, 148.0, 146.3, 131.8, 130.0, 126.8, 121.2, 118.4, 117.8, 111.5, 109.3, 56.0, 55.9, 53.9, 44.4, 28.1, 20.5; Spectroscopic data are in agreement with the published data [S2].

REFERENCES

[S1] Rueping, M., Vila, C., Koenigs, R. M., Poscharny, K., Fabry, D. C. Dual catalysis: combining photoredox and Lewis base catalysis for direct Mannich reactions. *Chem. Commun.* **2011**, *47*, 2360-2362.

[S2] Džambaski, Z., Bondžić, B. P. Dehydrogenative C(sp3)–H bond functionalization of tetrahydroisoquinolines mediated by organic oxidants under mild conditions. *Org. Biomol. Chem.* **2019**, *17*, 6420-6425.

[S3] Sud, A., Sureshkumar, D., Klussmann, M. Oxidative coupling of amines and ketones by combined vanadium- and organocatalysis. *Chem. Commun.* **2009**, 3169-3171.

[S4] Zhao, G., Yang, C., Guo, L., Sun, H., Chen, C., Xia, W. Visible light-induced oxidative coupling reaction: easy access to Mannich-type products. *Chem. Commun.* **2012**, *48*, 2337-2339.

[S5] Shen, Y., Li, M., Wang, S., Zhan, T., Tan, Z., Guo, C.-C. An efficient copper-catalyzed oxidative Mannich reaction between tertiary amines and methyl ketones. *Chem. Comm.* **2009**, *8*, 953-955.

[S6] Yu, J.-B., Peng, G., Jiang, Z.-J., Hong, Z.-K., Su, W.-K. Mechanochemical Oxidative Mannich Reaction: Evaluation of Chemical and Mechanical Parameters for the Mild and Chemoselective Coupling of N-tert-butoxycarbonyltetrahydroquinolines and Ketones. *Eur. J. Org. Chem.* **2016**, 5340-5344.

[S7] Wu, W., Wang, H., Chen, J., Bao, X., Tan, C., Ye, X. Dicyanopyrazine-derived Chromophore as An Efficient Photocatalyst for α-amino C-H Bond Functionalization. *Asian J. Org. Chem*. **2021**, 2876–2879.

[S8] Ruiz Espelt, L., Wiensch, E. M., Yoon, T. P. Brønsted acid cocatalysts in photocatalytic radical addition of α-amino C-H bonds across Michael acceptors. *J. Org. Chem*., **2013**, *78*, 4107–4114.

[S9] Murahashi, S.-I., Nakae, T., Terai, H., Komiya, N. Ruthenium-Catalyzed Oxidative Cyanation of Tertiary Amines with Molecular Oxygen or Hydrogen Peroxide and Sodium Cyanide: sp3 C−H Bond Activation and Carbon−Carbon Bond Formation. *J. Am. Chem. Soc.*, **2008**, *130*, 11005-11012.
